# Assessment of the safety of antimalarial drug use during early pregnancy (ASAP): protocol for a multicenter prospective cohort study in Burkina Faso, Kenya and Mozambique

**DOI:** 10.1186/s12978-015-0101-0

**Published:** 2015-12-04

**Authors:** Halidou Tinto, Esperança Sevene, Stephanie Dellicour, Gregory S. Calip, Umberto d’Alessandro, Eusébio Macete, Seydou Nakanabo-Diallo, Adama Kazienga, Innocent Valea, Hermann Sorgho, Anifa Valá, Orvalho Augusto, Maria Ruperez, Clara Menendez, Peter Ouma, Meghna Desai, Feiko Ter Kuile, Andy Stergachis

**Affiliations:** Clinical Research Unit of Nanoro, Institut de Recherche en Sciences de la Santé (IRSS), Bobo-Dioulasso, Burkina Faso; Eduardo Mondlane University, Maputo, Mozambique; Centro de Investigação em Saúde da Manhiça, Maputo, Mozambique; Liverpool School of Tropical Medicine (LSTM), Liverpool, UK; Departments of Pharmacy and Global Health, School of Public Health, University of Washington (UW), Seattle, USA; Medical Research Council, Fajara, The Gambia; Kenya Medical Research Institute Centre for Global Health Research, Kisumu, Kenya; Centers for Disease Control and Prevention, Atlanta, GA USA; Centers for Disease Control and Prevention, Nairobi, Kenya; Instituto de Salud Global de Barcelona, Barcelona, Spain

**Keywords:** Pregnancy, Malaria, ACTs

## Abstract

**Background:**

A major unresolved safety concern for malaria case management is the use of artemisinin combination therapies (ACTs) in the first trimester of pregnancy. There is a need for human data to inform policy makers and treatment guidelines on the safety of artemisinin combination therapies (ACT) when used during early pregnancy.

**Methods:**

The overall goal of this paper is to describe the methods and implementation of a study aimed at developing surveillance systems for identifying exposures to antimalarials during early pregnancy and for monitoring pregnancy outcomes using health and demographic surveillance platforms.

This was a multi-center prospective observational cohort study involving women at health and demographic surveillance sites in three countries in Africa: Burkina Faso, Kenya and Mozambique [(ClinicalTrials.gov Identifier: NCT01232530)]. The study was designed to identify pregnant women with artemisinin exposure in the first trimester and compare them to: 1) pregnant women without malaria, 2) pregnant women treated for malaria, but exposed to other antimalarials, and 3) pregnant women with malaria and treated with artemisinins in the 2nd or 3rd trimesters from the same settings. Pregnant women were recruited through community-based surveys and attendance at health facilities, including antenatal care clinics and followed until delivery. Data from the three sites will be pooled for analysis at the end of the study. Results are forthcoming.

**Discussion:**

Despite few limitations, the methods described here are relevant to the development of sustainable pharmacovigilance systems for drugs used by pregnant women in the tropics using health and demographic surveillance sites to prospectively ascertain drug safety in early pregnancy.

**Trial registration:**

NCT01232530

**Electronic supplementary material:**

The online version of this article (doi:10.1186/s12978-015-0101-0) contains supplementary material, which is available to authorized users.

## Background

Artemisinin-based combination therapies (ACTs) are among the most rapidly acting and effective antimalarials, providing life-saving benefits to adults and children. Almost all malaria-endemic countries have adopted ACTs as the first-line treatment for *P. falciparum* [1]. ACTs are recommended for the treatment of malaria during the second and third trimesters of pregnancy. However, artemisinin compounds are not recommended for use in the first trimester of pregnancy, unless there are no other drugs available, due to concerns about their potential embryolethal and teratogenic effects. With expanded access to ACTs, a growing number of women will likely be inadvertently exposed to an ACT in early pregnancy, during the period of organogenesis [2, 3]. While ACTs are not recommended for use during early pregnancy, pregnant women need safe and effective treatment and prevention measures to avoid the substantial adverse consequences of malarial infections to themselves and their developing fetuses. Adverse consequences of untreated malaria in pregnancy include severe anemia for women and low birth weight (LBW) for newborns, one of the principal causes of infant mortality in the African region [4]. Any risk of exposure to ACTs in early pregnancy must therefore be carefully weighed against the potential benefits of antimalarial treatment.

Animal reproductive toxicology studies of artemisinins demonstrate that embryotoxicity occurs through inhibiting human erythroid cell differentiation [5, 6], leading to either embryolethality [7] or other significant dysfunction in embryo hematopoiesis [8]. Extrapolation of this data to humans suggests that the embryo-sensitive period may be between 4 and 10 weeks post-conception [9], the period when the nucleated, metabolically active primitive erythroblasts predominate in the blood. However, among humans, the period of embryo-sensitivity is unclear, as the primitive erythroblasts are gradually replaced over several weeks by enucleated mature erythrocytes, which are less sensitive to the effects of artemisinins. In more recently conducted animal studies, no increase in toxicity was observed with the use of two artemisinin drugs or artemisinins plus other antimalarials [10, 11]. The limited safety data available from human observational studies of artemisinins is reassuring with no adverse effects on infants at birth or through their first birthday reported to-date [12–19]. While embryotoxic effects have yet to be observed in epidemiologic studies in humans, increased obstetric complications including fetal distress at birth, Caesarean section births and any adverse pregnancy outcome were observed in one study among women treated with artemisinins in pregnancy, specifically among primigravidae and older women [20]. Therefore, improvement of surveillance methodologies and ongoing assessment of the safety of ACTs in pregnancy remains important for malaria-endemic settings.

There have been multiple calls for generating more safety data on use of the artemisinins in early pregnancy in humans and for new approaches to obtain more systematically collected safety information during the post-marketing phase http://www.who.int/malaria/publications/atoz/9789241596114/en/. Pregnant women are routinely excluded from pre-licensure clinical trials for fear of harming the mother or the developing fetus, and thus most medicines, including ACTs, are marketed with very limited information on safety or efficacy during pregnancy. Although the World Health Organization (WHO) does not recommend the use of ACTs in the first trimester unless there is no alternative and the life of the woman is in danger, reports of antimalarial drug use among pregnant women in some settings show frequent use of ACTs [20–22]. This situation, medicines not recommended therefore not prescribed—not prescribed therefore no information, has rightly been described earlier as a “Catch-22” [23]. We previously estimated that with an average of 32 million pregnancies occurring annually in malaria endemic countries in Africa, an estimated 1 to 2 million pregnancies are exposed to ACTs during the embryo-sensitive period [24]. Without prescribing indications (except 2nd and 3rd trimester) or risk management programs for women of childbearing age (WOCBA) or women with suspected or confirmed pregnancy, the potential for inadvertent exposure to artemisinins during early pregnancy is high, and in many cases, unavoidable.

As part of a robust and sustainable approach to malaria case management, safe and effective antimalarial treatments must be identified for women during early pregnancy. Pharmacovigilance is needed to detect, report, and manage possible adverse effects of artemisinins on pregnancy outcomes. Improved methods for pharmacovigilance in early pregnancy are needed to inform the development of treatment guidelines and evidence-based practices. Herein, we describe a novel observational methodology using active surveillance techniques for pharmacovigilance of antimalarial use in early pregnancy, with an emphasis on assessing the effects of exposure to ACTs within health and demographic surveillance sites. This approach has been field-tested in three sites associated with the Malaria in Pregnancy Consortium (MiPc). Herein we provide an overview of the methods. The study results will be presented separately following completion of the study and data analysis.

## Goal and study aims

The goal of the Assessment of the Safety of Antimalarial Drug Use During Pregnancy (ASAP) study was to develop surveillance systems for identifying exposures to antimalarials during early pregnancy and for monitoring pregnancy outcomes. The specific aims were to develop and field-test active surveillance tools and procedures for identifying ACT exposure in early pregnancy and for assessing pregnancy outcomes, including assessment of major congenital malformations detected by a surface examination, miscarriages, stillbirths and live births. A further aim was to contribute data to inform the evidence-base and safety profile regarding the relationship between use of ACTs during early pregnancy and risk of adverse pregnancy outcomes such as miscarriages, stillbirths, and major congenital malformations. The protocol was reviewed and approved by the Ethical Review Boards of the Kenya Medical Research Institute (KEMRI), U.S. Centers for Disease Control and Prevention (CDC), National Bioethics Committee in Mozambique, Centre Muraz Institutional Ethics committee and National Ethics committee in Burkina Faso, Liverpool School of Tropical Medicine in the UK, and the Institutional Review Board of the University of Washington.

## Methods

### Study design

This multi-centered, non-interventional, prospective cohort study consisted of field activities at three MiPc partner sites in sub-Saharan Africa located in Siaya County, Kenya; Nanoro, Burkina Faso; and Manhiça, Mozambique. All sites were involved in developing and field-testing active surveillance methods for identifying exposures to antimalarials during early pregnancy; monitoring pregnancy outcomes; and assessing infant outcomes. Guided by a common protocol, each site implemented prospective observational methodologies within their operating health demographic surveillance system (HDSS) programs, allowing for active surveillance and records linkage for ACT exposures and pregnancy outcomes, with an emphasis on identifying first-trimester exposures to antimalarials. Moreover, all sites identified and recruited women as early as possible in pregnancy.

### Study sites

All three study sites are under health and demographic surveillance system (HDSS) and members of the International Network of field sites with continuous Demographic Evaluation of Populations and Their Health in developing countries (INDEPTH) [http://www.indepth-network.org/]. Through the HDSS, vital events such as pregnancies, births, migrations and deaths are monitored, and information on household and socio economic characteristics are collected through household visits carried out on regular basis. The HDSS provides a platform to identify women of childbearing age and pregnancies as well as to follow them through active surveillance of pregnancy outcomes.

#### Asembo - Siaya County, Kenya

Siaya is a rural community located in Rarieda District in Siaya County, lying northeast of Lake Victoria in Nyanza Province, western Kenya. The site is part of the KEMRI/ CDC Research and Public Health Collaboration HDSS which has been described in detail elsewhere. In this area, *P. falciparum* malaria transmission is perennial and holo-endemic with approximately 18 % of pregnant women being parasitemic at first antenatal clinic visit [25, 26]. The selected study area covered 33 villages and approximately 25,000 people under HDSS and enhanced morbidity surveillance since 2005 through the KEMRI/CDC International Emerging Infection Program (IEIP) to investigate major infectious disease syndromes [27]. As part of this population-based infectious disease surveillance project (PBIDS), trained fieldworkers visited households regularly (on a weekly basis from January 5, 2010 to May 26, 2011 and then bi-weekly from May 27, 2011 onwards) collecting information on all symptoms since the previous visit, the source of care and any medication taken, including specific antimalarial medication. All PBIDS participants receive free care for infectious symptoms at a referral health facility, Lwak Mission Hospital, where information on diagnosis and treatment is recorded using the HDSS ID for record linkage. The study was carried out in collaboration with PBIDS to enabling cross-validation of drug exposure data obtained through prospective home visits, health facility records and self-report during pregnancy.

#### Nanoro, Burkina Faso

The Nanoro Department is located in central Burkina Faso, West Africa, approximately 85 Km from Ouagadougou, the capital city. The study area is located within the HDSS catchment area and covers approximately 33,300 people living in 15 villages. Malaria is hyperendemic with seasonal transmission (July–November). It represents the main reason for consultations at health facilities all year round, with a peak between September and November. The disease represents a significant burden on the population, but especially pregnant women and children. *Plasmodium falciparum* is the main malaria species. The Nanoro HDSS was established in 2009 by the Clinical Research Unit of Nanoro (CRUN). It covers a size of 600 Km^2^ for a total population of 63,000 inhabitants in 24 villages (8 peripheral health facilities) [28]. In addition to the regular surveys, a continuous weekly monitoring is conducted by village reporters. The CRUN is located in the Centre Médicale Saint Camille de Nanoro (CMA), the referral hospital of the Nanoro health district [29]. A fully functioning clinical laboratory (parasitology, biochemistry, hematology, microbiology and blood/cells in vitro culture) and digital X-ray facility, have been set up with new equipment (all validated before their first use), including back-up equipment to ensure reliable services. Therefore most routine diagnosis for patients care management can be done locally on site.

#### Manhiça, Mozambique

The Manhiça District is a rural community located in southern Mozambique, 80 km north of Maputo city. The site is part of the Centro de Investigação em Saúde de Manhiça which collaborates with Eduardo Mondlane University, Mozambique, and the Centre for International Health, University of Barcelona, Spain. This site is well described elsewhere [30]. *P. falciparum* malaria is the most prevalent in the site and transmission is perennial with two well-demarked seasons, a dry season (May-October) and a rainy season (November-April). The entomological inoculation rate estimated in 2002 was of 38 infective bites/person/year [31]. In addition to the HDSS of approximately 92,000 inhabitants, the center has a morbidity platform to monitor information on illnesses and treatments for individuals under 15 years old through registries of all outpatient and inpatient visits to the health facilities in the HDSS area. This platform includes all five peripheral health facilities and the referral Manhiça District Hospital. The clinicians working on this platform are trained on malaria diagnosis and case management and to record all visit including information on diagnose, laboratory results and treatments prescribed. A basic laboratory facility is available for blood smear and hemoglobin (using Hemocue). A standardized form is used for outpatient and inpatient, data is recorded in a database and available for record linkage. As part of this study, the morbidity platform was extended to include women in childbearing age (15–49 years).

### Training

Training was critical to support high quality data and standardization across sites. In addition to the overall protocol and ethics training, nurses from all sites were trained on newborn examination for recognition of congenital malformations. They were also trained in the use of Ballard score as well as ultrasound for gestational age assessment. The nurses were informed that the purpose of the newborn examination was to identify certain problems the baby may have been born with, and therefore provide each baby with the best appropriate care available; and to document the occurrence of congenital malformations, or birth defects, in each country, which can help in understanding the safety of medications given during pregnancy. Participants were also urged that the success of the project depended on the quality of the examiner’s work and that having a routine order of examination makes it less likely to forget any parts of the examination. The ultrasound training was targeted to the nurses from the antenatal clinic and focused on pregnancy identification, assessment of fetal vitality and gestational age calculation. For pregnancy identification, the trainees had to identify gestational sacs, embryos or heart beats and for gestational age assessment they were trained on measurement of crown‐rump length, biparietal diameter, head and abdominal circumference and femur length using standard training manuals developed by the Malaria in Pregnancy Consortium (see Additional file 1 and Additional file 2).

### Community sensitization

Our first step involved community sensitization through meetings held at each location with community leaders aiming at informing them about the planned study and providing a forum for questions and clarification. This was an important step to ensure good participation, particularly when addressing potentially sensitive subjects such as pregnancy in Africa. Indeed previous studies on malaria in pregnancy have highlighted the importance of the social context for the uptake of interventions [32]. Therefore researchers should take into account the cultural and ethical norms of the population(s) under study. Sensitization of the community for early detection of pregnancies and early presentation at ANC was conducted by social scientists. Pregnant women were also informed about the availability of pregnancy tests and ultrasound for pregnancy diagnosis at ANC. Printed informative material was distributed through community meetings.

### Enrollment into the study

Guided by an overall study protocol, the three individual field sites developed novel enrollment and registry practices to create infrastructure for surveillance of medications used in pregnancy. Eligible participants consisted of pregnant women residing in the defined catchment areas of each site, who planned to remain in the study area through delivery and who were willing and able to provide written informed consent. Exclusion criteria across all study sites were refusal to participate or be followed up at the end of pregnancy and any condition that would interfere with the ability to provide written informed consent or provide an accurate medical history. In Mozambique and Burkina Faso, pregnant women were identified within the health demographic surveillance system (HDSS) through repeated surveys as described above. In addition, field workers visited village reporters on weekly basis to learn about any pregnancy identified in their community. All identified pregnant women were invited to the ANC and assessed for eligibility. Baseline information was then collected and the data entered into a pregnancy register. Records linkage with data collected at outpatient and inpatient visits using electronic data capture method was also used in the two sites for identify possible exposure to ACTs during the first trimester of the pregnancy. In Kenya, recruitment was broadened to include women of childbearing age participating in the PIBDS program described above in order to detect pregnancies as early as possible through community based strategies. Study staff visited all homes in the PBIDS and enrolled consenting women of childbearing age who were assessed for pregnancy at the time of enrolment and again approximately every 3 months thereafter. Any participant with a detected pregnancy was referred to the antenatal clinic at a study health facility where trained study nurses would confirm the pregnancy and offer free ANC. Additionally, all eligible, consenting women in ANC at the study facility were assessed and enrolled.

At enrollment, each site collected detailed demographic data, information on possible risk factors and potential confounders via a detailed questionnaire administered to enrolled participants at first ANC visit, any antenatal follow up visit and at the end of pregnancy. Table 1 presents the sources of information for drug exposure and pregnancy outcomes by study site. Demographic and socioeconomic data, history of malaria episodes, co-morbidities, other medication use (including herbal or traditional remedies) during pregnancy, and obstetric history were collected from all participants.

**Table 1:** Summary of ascertainment of drug use approaches by study site

Event	Source	Sites		
		Asembo	Manhiça	Nanoro
Drug exposure	Retrospective Self-report	√	√	√
	Health Facility Treatment records	√	√	√
	Outpatient visit database		√	
	Prospective Self-report	√		
Pregnancy outcomes	Health facility assessment	√	√	√
	Home base assessment	√		√

### Ascertainment of antimalarial drug exposures

The ascertainment of drug exposure was multi-modal and included self-report (prospective and retrospective) and linkage to treatment records at local health facilities, drug prescribing and dispensing clinics. The following definitions were used to classify “possible” ACT exposure as exposure identified in only one data source and “confirmed” ACT exposure as exposures identified in 2+ data sources (for example self-report during interview at ANC visit and confirmed in health facility outpatient records).

#### Drug identification

Before study initiation an assessment of antimalarials available in the study areas was conducted and country-specific visual aids and pictorial keys were prepared. These tools were used to assist the participants recall drug names during interviews. Study staff reviewed participants’ drug packages or tablets when available, as well as reviewed and abstracted prescribing information from ANC cards, where available. Data collected included timing of exposure, medication name, dose, and duration.

#### Retrospective self-reported drug exposure

Subjects were asked about exposure to antimalarials and other medications currently used and used earlier in their pregnancy at a baseline interview conducted by study staff. Subjects were also asked about exposures at an ANC clinic visit after pregnancy status was confirmed as well as at all ANC visit and during pregnancy outcome follow-up visits. The timing of possible drug exposures and possible peri-conceptional exposure was dated retrospectively using estimated gestational age. Medication use that was self-reported to have been prescribed or dispensed at the health facility was verified and matched with drug prescription records at health care facilities, clinic databases of drug prescribing and dispensing, ANC clinic visit records, and records from delivery units where possible.

#### Health facility records drug exposures

Records on drugs dispensed at health facilities located within the study catchment areas were extracted from the health facility log-books, from the morbidity surveillance database and/or systematically abstracted from outpatient department records.

#### Prospective self-report drug exposures

The PBIDS program in Kenya offered a unique opportunity to collect prospective detailed data on drug exposure and morbidity on a weekly and then bi-weekly basis at home, which limits recall bias and potentially improves specificity in reports of the timing of drug exposure.

### Gestational age assessment and classification

The accurate assessment of gestational age was essential to identify drug exposures occurring during the embryo-sensitive period and avoid misclassification of exposure. Gestational age was assessed via multiple methods, as available, including date of last menstrual period, Ballard Score, fundal height and ultrasound (Table 2). Study staff were trained on newborn examination for Ballard Score assessment as well as fundal height measurement. They were also trained on the use of ultrasound dating scans using portable ultrasound machines, i.e., *Sonosite 180 plus,* Sonosite Inc. USA. Women were encouraged to attend ANC as early as possible where free ultrasound scans were offered to participants presenting before 24 weeks of gestation [33, 34]. The study nurses were trained on identification of the pregnancy, assessment of gestational age and to refer any suspicion of abnormality for better assessment to a physician. Quality control for ultrasound scans was implemented following the ultrasound training of the study nurses. All ultrasound images were downloaded from the machine to a laptop and a random sub-sample (20 %) were sent after removing all identifiers from the images to a consulting ultrasound radiologist located at the University of Washington. This reviewer provided feedback to the midwives highlighting areas needing re-training or special attention. If required, additional training materials were sent for review with the study nurses. Additionally, fundal height was also measured at each ANC visit at the study health facilities and recorded by a trained study nurse and information on last menstrual period was collected. Ballard score was performed and dated by trained health care providers at all sites within 7 days of birth.

**Table 2:** Accuracy of gestational age assessment by method

Method of Gestational Age Assessment	Accuracy Correction^a,b,c^	Accuracy Scale^d^
Ultrasound before 14 gestational weeks	±5 days	1 (most accurate)
Ultrasound between 14 and 28 gestational weeks	±2 weeks	2
Ultrasound after 28 gestational weeks	±3 weeks	3
Ballard score within 7 days of birth	±2 week	4
LMP	±4 weeks	5
Fundal Height	±6 weeks	6 (least accurate)

^a^[37]

^b^[38]

^c^[39]

^d^Gestational age was be assessed using the most accurate technique available

### Pregnancy outcomes assessment

Pregnancy outcomes of interest consisted of miscarriages, elective terminations, fetal deaths/stillbirths, low birthweight, small for gestational age, and live births. Fetal resorption and spontaneous abortion were clear safety signals from pre-clinical studies in all species studied and miscarriage was therefore an important endpoint to consider for the risk-benefit evaluation of ACTs. All participants were encouraged by study staff to deliver at the nearest health facility, and both health facility-based and home-based follow-up systems were used to ascertain pregnancy outcomes, as home birth remained common in all three sites. For home-based follow-up, traditional birth attendants (TBAs) were instructed to alert study staff of the occurrence of any pregnancy outcome, including miscarriage, stillbirth or live birth. Following a home delivery, study staff visited the household as soon as possible [48 h in Burkina Faso, up to the 6 weeks postnatal appointment (post-partum visit) in Mozambique and no cut off were specified in Kenya] following the event to administer a standard questionnaire and examine the newborn (including live births and stillbirths where possible). Study staff were blinded to the exposure status of women, and trained to systematically collect pregnancy outcomes and conduct newborn examinations using a “newborn surface examination manual” produced by MiPc. All information collected including vital status of the baby, sex, head circumference, length and weight was recorded on a pregnancy outcome case report form (CRF). The date, place and time of examination, and the age of the baby at examination (number of days since birth) were also collected, so that analysis can separate babies assessed at birth from those assessed some time later. Any abnormalities observed and any adverse outcomes in the mother or baby were noted and reported in the CRF.

A systematic surface examination based on the MiPc standard manual (see Additional file 1) was carried out to identify congenital anomalies which are visible to the examiner and do not require specialized staff or equipment. Digital photographs of abnormalities were taken for the purposes of confirmation and classification. Cases of suspected malformation or abnormality were reviewed by a study pediatrician for confirmation. Cases needing further investigation or intervention were referred to the appropriate facilities/ specialist.

## Sample size determination

The required sample size was determined using the minimum relative risk (RR) to be detected and the frequency of adverse pregnancy outcomes of interest in the non-exposed or comparison population. A summary of number of exposed and unexposed pregnancies to be recruited per site are provided in the Table 3.

**Table 3:** Summary of number of exposed and unexposed pregnancies to be recruited per site

Sites	Approximate Population Size	Approximate Pregnancies per year	Projected number of exposures per year^a^	Sample Size over 1 years of recruitment^b^		Ratio of exposed to unexposed
			Embryo sensitive period 6 weeks (exposure risk %)	Fully documented Exposed	Fully documented Unexposed	
Kenya	25,000	1085	3 %	14	458	1:30
Burkina Faso	30,000	1000	6 %	42	658	1:16
Mozambique	33,000	1000	6 %	42	658	1:16
Overall	88,000	3085		98	1768	

^a^This was based on a number of assumptions, as follows: 1) the average pregnancy is 266 days (38 weeks) from conception (280 days or 40 weeks from LMP), 2) the average number of treatments with ACTs in adults in the study areas is approximately 0.5 treatments per year (1 every 2 years), and 3) the total fertility rate is estimated at 5.5 [29]. Under these conditions, we estimated the probability that an embryo would encounter artemisinins inadvertently during the critical 42 day (6 weeks) period of its development (week 4 to week 9 inclusive, from conception) is about 6.0 %. In the absence of regular pregnancy testing to exclude early pregnancy, the potential ratio of exposed versus unexposed pregnant women is estimated at 1:16. Exposure risk will be lower for the Asembo site as some women will be detected early and will be counselled not to take ACTs in the first 3 months of pregnancy

^b^Estimating that about 70 % of exposures can be documents reliably and followed up to pregnancy outcome

The ratio of exposure to antimalarials versus non-exposure in early pregnancy was estimated to be approximately 1:16 in Mozambique and Burkina Faso and 1:30 in Kenya where the probability of inadvertent exposure would be reduced significantly due the introduction of pregnancy testing at the household level. This was based on a number of assumptions, as follows: 1) the average pregnancy is 266 days (38 weeks) from conception (280 days or 40 weeks from LMP), 2) the average number of treatments with ACTs in adults in the study areas is approximately 0.5 treatments per year (1 every 2 years), and 3) the total fertility rate is estimated at 5.5 [30]. Under these conditions, we estimated the probability that an embryo would encounter artemisinins inadvertently during the critical 42 day (6 weeks) period of its development (week 4 to week 9 inclusive, from conception) is about 6.0 %. In the absence of regular pregnancy testing to exclude early pregnancy, the potential ratio of exposed versus unexposed pregnant women is estimated at 1:16. However, in Kenya, we anticipated that the probability of inadvertent exposure would be reduced significantly, to approximately 3.0 %, as pregnancy would likely be detected earlier and therefore less inadvertent exposure would result. We therefore expected the ratio of exposed to unexposed in Kenya to be approximately 1:32.

### Miscarriages

A conservative estimate is that only 50 % of the pregnancies will provide sufficient information to contribute to the analysis due to refusal to participate, late recruitment, loss to follow-up, uncertainties about gestational age assessment or unreliable data on drug exposure. The background rate of clinically recognized miscarriages (from 6 to 28 weeks post-LMP) is estimated at 12.2 %. There are no published data on background rates of miscarriage for the study area or overall for Kenya, however, it is likely that a significant proportion of early miscarriages could be missed (especially between 6 to 9 weeks gestation). If one assumes that only two-thirds of the miscarriages can be captured (8.1 %), a sample size of 466 documented pregnancies (14 exposed and 452 unexposed women) was calculated to have 80 % power to exclude a 3.1 fold increased risk of spontaneous abortions from 8.1 % background rate to 25.2 % in exposed pregnancies (1-sided alpha level of 0.05).

### Congenital malformations

Because information on background rates of major malformations is not available for Africa, the background rate in the unexposed control group was based on estimates from the USA [35, 36]. The anticipated background rate of major malformation detectable at birth is approximately 2 % in western populations (and 3 % by 5 years of age), but this estimate includes major malformations that are not easily detectable by non-specialized staff (e.g., cardiac malformations). The rate of major congenital malformations among 69,277 live-born and stillborn infants and elective terminations at Brigham and Women’s Hospital in Boston was 2.2 % at birth [36]. Of these, about 0.6 % due to chromosomal abnormalities and genetic disorders, and the remaining 1.6 % were due to non-genetic defects. Of the non-genetic defects, 0.7 % was birth defects which are not easily detectable at birth without specialized staff and equipment (such as heart defects) [35]. Therefore, we restricted the assessment at birth to visual inspection and a surface examination, conservatively anticipating that the prevalence of major malformations detectable at birth would be approximately 0.9 %. Table 3 shows the sample size based on the estimated number of live-births and stillbirths in the 3 study sites if likelihood of ACT exposure was 6 % in Burkina Faso and Mozambique, and a 3 % exposure risk in western Kenya. All sites assume 0.5 treatments with ACTs in an adult population and a fertility rate of 5.5. A sample size of 3085 women was estimated to contribute 1866 (70 %) documented pregnancies to the analysis (98 exposed and 1768 unexposed) after accounting for possible attrition (i.e., loss to follow up, unreliable gestation or exposure data). With this minimum estimated sample size, our study was 80 % powered to detect up to a 5-fold increased risk for ‘any’ major congenital malformations with a one-sided alpha level of 0.05.

## Discussion

The goal of this study was to develop surveillance systems for identifying exposures to antimalarials during early pregnancy and for monitoring pregnancy outcomes. Although the methods described herein could contribute to provide estimates of reassurance about the safety profile of ACTs in early pregnancy, it presents several limitations: Indeed, only the Kenya site where an active surveillance system was carried out will allow the detection of early miscarriage as well as the early identification of pregnancy. However although the active surveillance system is more adequate for such detection/identification, it is unlikely that a national pharmacovigilance center or program in Africa can easily implement such methods in their policies’ routine practices as this will require more resources that typically available without external support. Moreover, once detected, the methods does not allow an easy assessment of induced abortions (illegal in many Africa countries unless the life of the mother is at risk) which could have been misclassified as miscarriage or be loss to follow up. However induced abortions are unlikely to differ between exposure groups and therefore bias effect estimates. As another limitation, the methods described here do not allow the differentiation between confirmed and unconfirmed malaria. This is particularly important in Africa where a high percentage of women might be exposed to malaria. In such context, it appears difficult to know the ‘’true control group” without knowing the malaria infection status of the non-ACT exposed pregnant women. Finally, the surface examination system used in this study does not allow the detection of non-visual congenital abnormality such as heart defects.

## Conclusion

Despite the few limitations listed above, this prospective observational approach has the potential to contribute additional data to inform the safety profile of ACTs in early pregnancy, and inform the benefit-risk profile of ACTs in similar populations. The optimal methodology for drug safety surveillance in pregnancy in resource-limited settings will likely vary based on local needs and available resources; including available methods for gestational age determination, possibility of community-based pregnancy detection, assessment of pregnancy outcomes, measurement of antimalarial exposure, and confirmation of malaria cases. Our active surveillance approach to pharmacovigilance in early pregnancy using HDSS platforms can contribute tools for use or adaptation in other settings, as well as serve as a resource to inform the development of methods to identify signals and evaluate potential risk from drug exposure in early pregnancy.

## Additional files

Additional file 1:
**Newborn Examination.** (PDF 1203 kb) Additional file 2:
**Ultrasound Reference Manual for Pregnancy Dating.** (PDF 3017 kb)
