# Supplementary material for: Assessment of the safety of antimalarial drug use during early pregnancy (ASAP): protocol for a multicenter prospective cohort study in Burkina Faso, Kenya and Mozambique
Source: Reprod Health. 2015 Dec 4;12:112. doi: 10.1186/s12978-015-0101-0 (PMC4670540; doi:10.1186/s12978-015-0101-0)
Supplement: Additional file 1: — Newborn Examination. (PDF 1203 kb) [file 12978_2015_101_MOESM1_ESM.pdf]

Newborn Examination

Training Manual

With thanks to:
Claudia Turner for some of the slides from her SMRU Newborn exam presentation
Dr. Janelle Aby, Stanford School of Medicine, for many of the pictures which can be found at
http://newborns.stanford.edu/PhotoGallery/
Dr. François Luks, Dept. of Pediatric Surgery, Brown Medical School,
http://bms.brown.edu/pedisurg/Brown/IBCategories.html,
Dr. Paul D. Larsen, University of Nebraska Medical Center and Dr. Suzanne S. Stensaas,
University of Utah School of Medicine,
http://library.med.utah.edu/pedineurologicexam/
and Sadie Rae Litner for her many appearances in the pictures.

Table of Contents

I. INTRODUCTION 4
- Newborn examination 4
- Congenital malformations 4
- Purpose of the newborn examination 5
- Importance of the examiner 6
- Every baby needs an examination 6

II. COMPLETING THE NEWBORN EXAMINATION 8
- The order of the exam: 8
- Observation 8
- Color 9
- Measurements 9
  - Respiratory rate 9
  - Head circumference 9
  - Length 10
- Head and skull bones 10
- Face 13
- Chin 13
- Mouth and palate 14
- Nose 15
- Ears 15
- Eyes 15
- Chest 16
- Abdomen 16
- Limbs 19
- Limbs 20
- Genital exam - Male 22
- Genital Exam – Female 24
- Anus 24
- Back & Spine 25
- Neural Tube Defects 26
- Skin 27
- Birthmarks 28
- Neurological Examination 29

III. SUMMARY 29

IV. TRAINING 29

V. TRAINING LOG 30

VI. APPENDIX 31

I. INTRODUCTION

A study is being conducted at this clinic to better understand certain issues regarding malaria during pregnancy. Some of these issues involve understanding which drugs are best to use to treat malaria during pregnancy and looking at the safety of these drugs when used at different times during pregnancy.

Malaria occurs frequently in this community. During pregnancy, a woman's immune system is weakened, making her more susceptible to malaria infection, as compared with women who are not pregnant. Malaria in pregnant women can cause several complications that are dangerous to the mother and her unborn child, including severe maternal anemia, hemorrhage, maternal death, miscarriage, stillbirth, and low birth weight of the newborn.

Newborn examination

As part of this project, you will be conducting a newborn examination. All births (both live and still births) that occur within the study will need to have a newborn exam completed. A newborn examination has many purposes including:

- It allows us to quickly identify certain problems the baby may have been born with, and therefore provide each baby with the best appropriate care available; and
- It allows us to document the occurrence of congenital malformations, or birth defects, in each country, which can help us to understand the safety of medications given during pregnancy.

Congenital malformations

A congenital malformation, otherwise known as a "birth defect," encompasses a wide variety of conditions and is most simply defined as a defect which is present at birth. Birth defects may be easily seen, such as a cleft lip, or may affect internal organs and be more difficult to detect without sophisticated testing equipment, such as with certain heart defects. Birth defects can be cosmetic only, such as a dark birthmark on the face, or they can be life threatening such as a baby born with their intestines on the outside of the body. Our work will be focused on identifying only those birth defects which can be seen when looking at the external anatomy of the newborn.

In industrialized countries such as the United States and Europe, approximately 3% of all babies born have a major malformation which is present at birth. This is known as the background rate, which is a measure of how frequently birth defects are known to occur within a country. In industrialized countries, all babies are examined shortly after birth and their results are recorded, providing us with a count of the number of babies which are born abnormally. In most developing countries, we do not know how frequently babies are born with defects because newborn examinations either do not occur routinely, or if they do occur, the results are not recorded anywhere. Therefore, we do not know if birth defects are more common or equally as common in developing countries compared to industrialized countries.

There are several causes of birth defects, most frequently:

- Genetic defects such as Down syndrome - account for 10-15% of all birth defects
- Teratogens -7-10% of all birth defects
    - A Teratogen is any agent that interferes with normal embryonic development such as alcohol, X-rays, medications such as thalidomide or infectious diseases like rubella.
- Multi-factorial, ie. due to the interaction of several factors- 30-35%

Up to 50% of birth defects still have an unknown cause.

For the purposes of this project, it is not important to be able to correctly name the different types of malformations. Rather, it is most important to be able to determine if a malformation is present. It is your job to identify the babies which appear abnormal. Confirmation of the abnormality and applying the correct name to a birth defect can be done later by a trained physician, or the official study examiner assigned to your clinic. If you are unsure if a baby has a malformation, that's okay, it is best to send that baby for further examination by the official study examiner in your clinic and let them decide if the baby is normal or not. Please take a picture of any possible abnormalities, so that they can be evaluated by the safety oversight group. Make sure that you include the baby's study ID number on the picture, so we will know which baby the picture belongs to.

Purpose of the newborn examination

Historically, there are several medications that women were given during pregnancy that were later found to cause harm to the baby. Whenever new medications are released into the market, it is always possible that pregnant women may:

- Use these drugs before they realize they are pregnant, or
- Use these drugs before the drug safety during pregnancy is fully understood.

Currently, the limited data on use of Artemisinin Combination Therapies (ACTs) for the treatment of malaria during pregnancy suggests that ACTs are safe for the unborn baby when used during the later part of pregnancy (in the 2nd and 3rd trimesters). However, we still need to improve the evidence by documenting both the number of normal babies born and the number of babies born with malformations among women who used antimalarial drugs during pregnancy. Considering the high frequency of malaria disease during pregnancy, women need to know that the drugs they are being offered are safe for their unborn baby. This research will help doctors and women make informed decisions about using drugs to treat malaria during pregnancy

How can we determine if a drug is safe for use during pregnancy? Before new drugs are released, they go through a series of clinical trial phases. The last phase is testing in humans. However, pregnant women are excluded from most clinical trials in an effort to protect the unborn babies from unknown effects of the drug. When pregnant women are included, clinical trials are often not large enough to detect certain problems that occur very rarely, such as birth defects. In order to know if pregnant women who take a certain drug are more likely to have a birth defect compared to pregnant women who did not use that drug, we first have to know what is the baseline frequency of birth defects in that population or country. This research is important because we will be able to establish background rates for birth defects which are

currently unknown in your country. We will then be able to compare these background rates to the rates of birth defects observed among pregnant women using different drugs to understand if women taking the drugs have a higher rate of birth defects.

Importance of the examiner
The success of the project depends on the quality of the examiner's work. Your work in conducting and recording these newborn screening exams is the most important part of being able to understand the safety of ACTs used during pregnancy. Your careful observations will allow us to correctly count the number of birth defects, an important number which is currently unknown in your community.

Every baby needs an examination
Why does every baby need an exam? Because if you don't look for an abnormality, you may not see it.

Many obvious defects are often missed simply because nobody looked carefully at all parts of the newborn. For example, a defect such as an imperforate (closed) anus is life threatening and might otherwise go unnoticed if not specifically checked.

Fortunately, because most of the babies are born without malformations, the newborn exam is fast and easy. All babies must have the exam completed and documented on the correct form, even if they appear normal. Filling in all of the boxes will provide the appropriate evidence that these babies were born normally. It is critical to the project that all boxes are filled in and all babies be examined the same way by everyone.

Even among babies born without a birth defect, the newborn examination can also help us determine if there is a problem with the baby such as one caused from infection, poor feeding, respiratory problems, or other abnormalities. The newborn examination provides the earliest possible detection of abnormalities or other serious problems. The examination also establishes a baseline for subsequent examinations. For example, is a certain condition or feature getting better or worse?

- **When to conduct the newborn examination:**

For babies born in the clinic, it is best to conduct the exam before the baby is sent home. For babies born at home, an effort should be made to complete a newborn exam within the first week of life. If an exam is not done within the first week of life, the baby should have a complete exam at their first visit. In all circumstances, the timing of the examination and age of the infant should be recorded on the appropriate form.

- **Length of the exam:**

A well skilled examiner can complete the surface exam within 5-10 minutes. However, when starting out, the exam may take a little longer as you get used to the order of the exam, filling in the forms, and all of the exam components.

- **Where to conduct the newborn examination:**

The exam is most quickly and easily performed on an exam table. You should examine all exposed body parts first with the infant's clothing on, then remove all the clothing and thoroughly examine the rest of the body.

Tell the mother that you will be examining the baby and take the baby to the examination area. If the mother wishes, she may watch you during the exam.

Before starting the exam, always wash your hands. Begin the exam when the baby is calm; this will help to take accurate measurements.

You will need a few materials in order to complete the exam:

- Tape Measure
- Digital Scale
- Examination Form
- IF available, stethoscope for auscultation (listening) to the heart and lungs.

Remember, babies cannot regulate their own body temperature and can quickly become too cold. After the examination is complete, remember to properly wrap the baby before returning them to their mother.

Newborn examination sheet

Some of the measurements you will take during the exam will be recorded on the "Newborn examination sheet", such as:

- Date and time of examination
- Weight (grams)
- Head Circumference (HC) (cm)
- Length (cm).

II. COMPLETING THE NEWBORN EXAMINATION

The exam will cover the following:

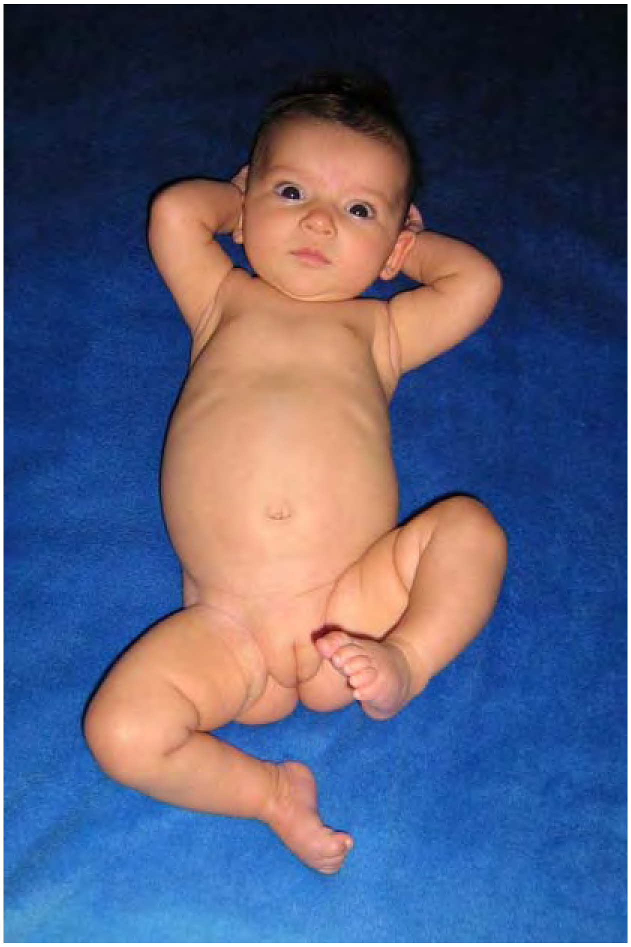

The order of the exam:

1. Record date and time of exam
2. General assessment and measurements
3. Skull
4. Face
5. Mouth & palate
6. Nose
7. Ear
8. Eyes
9. Chest
10. Abdomen
11. Arms
12. Hands
13. Legs
14. Feet
15. Genitals
16. Anus
17. Spine
18. Skin

Having a routine order of examination makes it less likely that you will forget any parts of the examination; however, your routine should be flexible. If the infant is quiet and relaxed when first approached, the assessment of respiratory rate and examination of the abdomen should be done before the baby is disturbed.

Observation

Before starting the exam take a minute to observe the baby. Ask yourself the following questions:

- What is the baby's color?
- In general does the baby look ill or well?
- Is the baby normally active?
- Is the cry normal?
- Are there any obvious malformations?
- Is the baby funny-looking, such as with a genetic syndrome like Down Syndrome?

Color

Notice the color of the newborn. Is he/she:

- Pale
- Cyanosis (bluish)
  - Perioral cyanosis is bluish discoloration around the mouth
  - Acrocyanosis is bluish discoloration of the feet and hands
- Jaundice (yellow)
- Normal

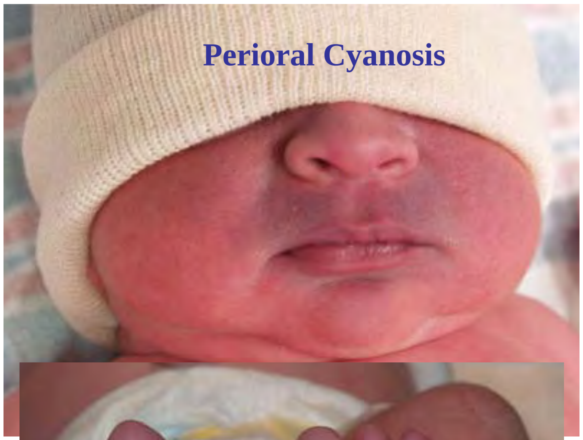

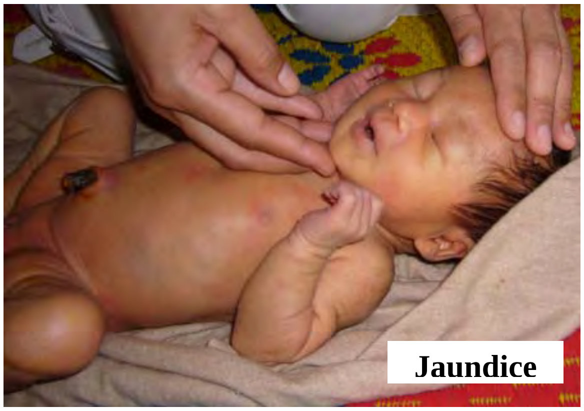

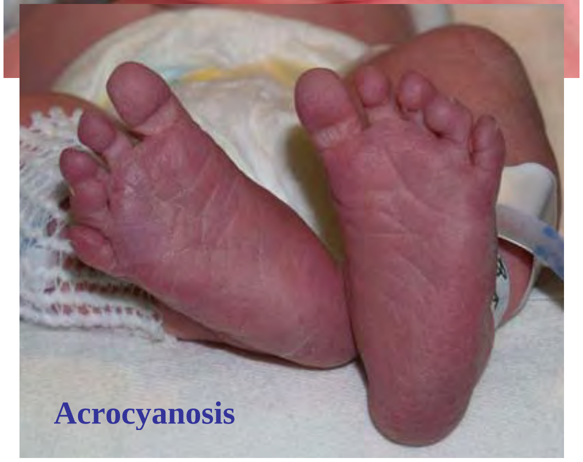

Measurements

Respiratory rate

- Normal range of a newborn is 40-60 breaths per minutes.
- Count the respirations for a full 60 seconds by watching the rise and fall of the chest. This should be done with the infant's chest exposed.
  - Counting respirations for 15 seconds and multiplying by 4 provides an inaccurate measurement in newborns. Make sure to count for the entire 60 second cycle.
- Take the respiratory rate when the infant is quiet. Remember to record the value on the appropriate form.

Head circumference

- Measure the head circumference in centimeters by placing the tape measure around the widest part of the head. This should be above the eyebrows and ears.

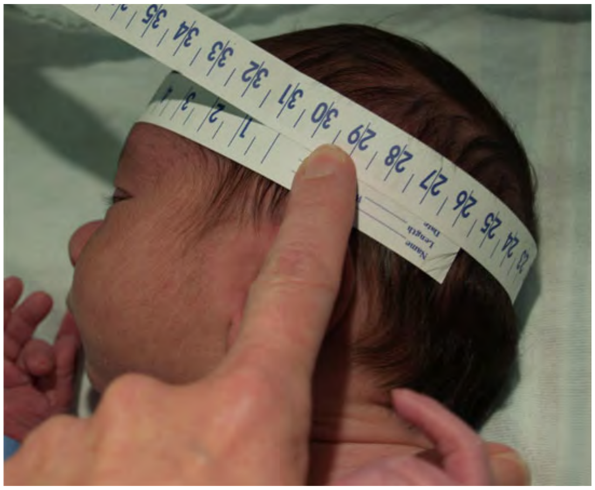

Length

- Measure the head-to-heel length in centimeters while the baby is fully extended. This can be difficult depending on how active the baby is. This is best done using a UNICEF measuring board, which has a base at the bottom. The baby's head is placed at the board (at 0 cm), and the baby's legs are pulled straight. The length is the distance between the top of the head and the bottom of the feet,
- Another method for measuring the length is to make a mark on the sheet at the top of the baby's head and then without moving the body, stretch the legs straight and make a mark at the bottom of the feet. The distance between the 2 marks can then be measured once the baby is moved. You must make sure that the tape measure or measuring stick is not held at an angle, which will result in an incorrectly long measurement.

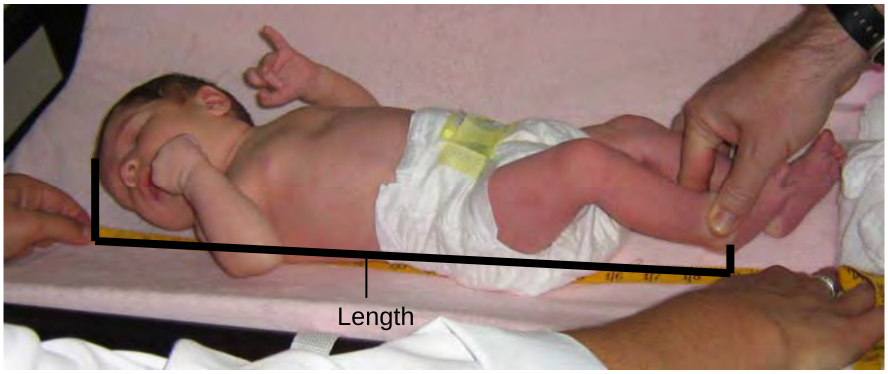

Head and skull bones

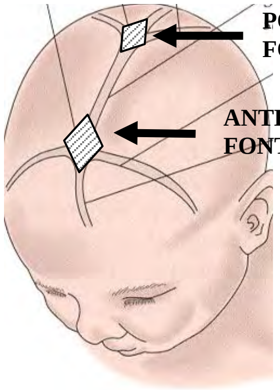

Fontanelle

The soft spot on the top of the baby's head is called the fontanelle. There is both an anterior and posterior fontanelle.

The anterior fontanelle can be:

- Normal
- Raised or bulging- suggests infection
- Depressed or sunken- suggests dehydration
  - If you think the anterior fontanelle is NOT normal, notify a clinician.

Sunken fontanelle

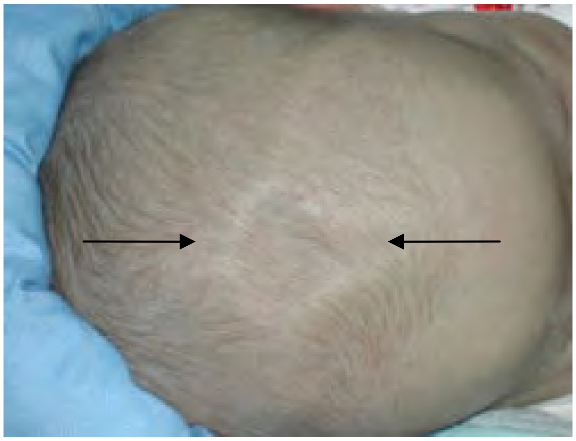

Photo credit Johann Dréo

Bulging fontanelle

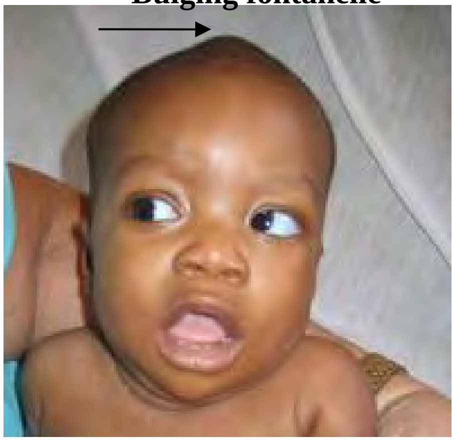

www.pmmdaily.blogspot.com/

Skull bones

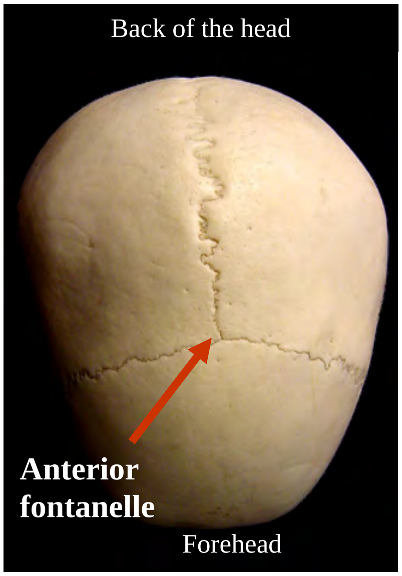

This is a photo of the skull bones from above (looking directly at the top of the head); the suture lines show where the bones fused after birth. While the baby is in the womb, these bones remain open (unfused) which allows the baby's head to compress when passing through the mother's pelvis. Sometimes, these bones can grow together too early which can result in an abnormal shape of the head.

When examining the skull bones and suture lines, you should be able to feel the large soft spot in the middle (anterior fontanelle) and a smaller soft spot further back on the head (posterior fontanelle). It is normal to feel a line where the bone is open between these two soft spots, as well as other ridges where the bones are open to allow the baby to pass through the birth canal. If these bones are already joined at the time of birth, this is called craniosynostosis and will usually result in an abnormally shaped head. If you suspect craniosynostosis, this should be noted on the form. Or, if you observe the baby to have an abnormally shaped head this should be noted on the form.

Molding and Caput

Molding means the head becomes misshapen by the pressure in the birth canal. When the mother is in labor (especially if it is a long labor) the baby's head may undergo molding or may develop swelling. Swelling of the scalp due to the pressure in the birth canal may also occur, and is called "Caput". Both of these resolve without treatment and do not need to be recorded.

Normal molding -

These two photos are examples of normal molding of the head after passing through the birth canal. This will resolve in a few days and does not need to be recorded.

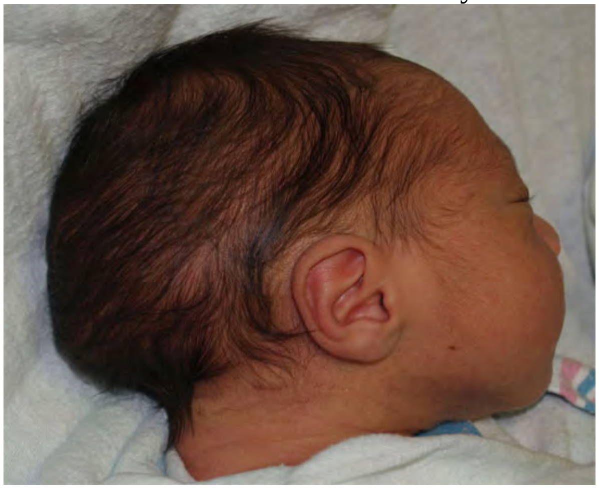

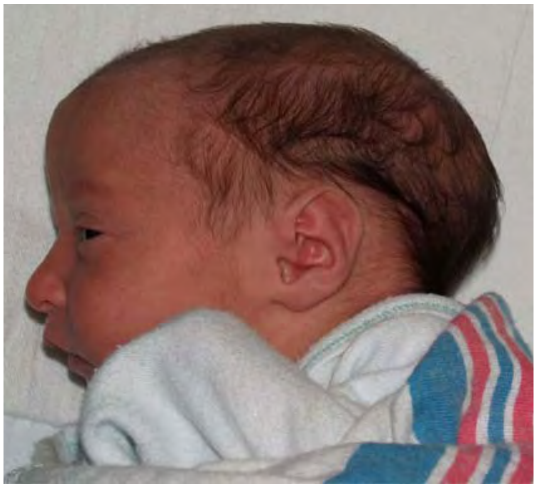

Caput

When a baby has caput the scalp can become filled with fluid which is known as scalp edema. If pressure is applied to the scalp in a baby with caput, you will see the characteristic pitting shown in the photo. Caput is normal and does not need to be recorded.

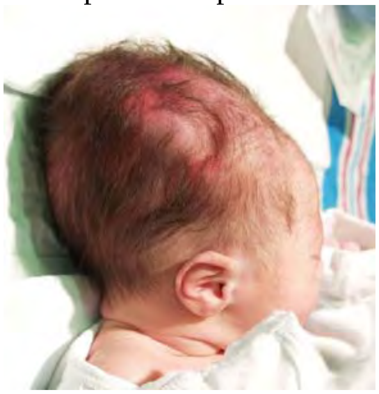
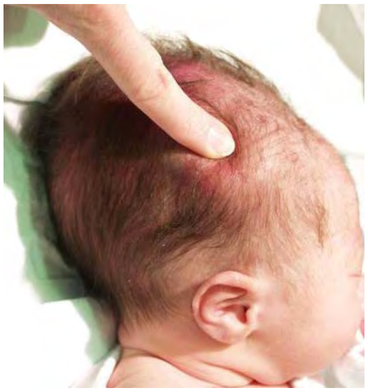
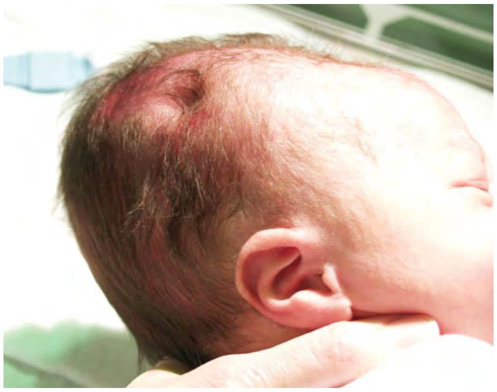

Cephalohematoma

Cephalohematoma is a collection of blood which accumulates on the scalp and can result from trauma during birth. A cephalohematoma never crosses suture lines, and so is never present in the midline of the head (note the dip in the middle). It will feel fluctuant on palpation. This does not need to be recorded.

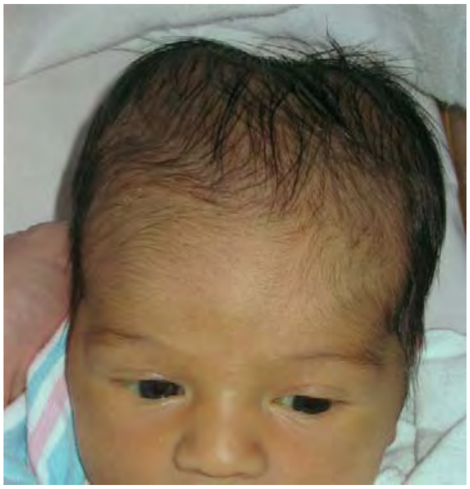

Hydrocephalus

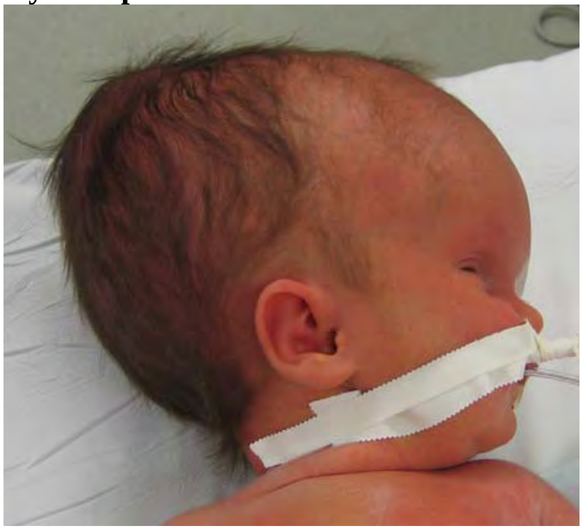

Hydrocephalus is an abnormal condition where cerebrospinal fluid collects in the ventricles of the brain which makes the head larger than normal. This should be marked on the form as part of the head examination.

Photocredit: David Wrubel, MD

Bruising

Bruising can occur to the head or face during delivery. It can be commonly seen as a consequence of difficult deliveries such as those babies born with a vacuum or forceps delivery. You may observe bruising to the head or face - this does not need to be recorded.

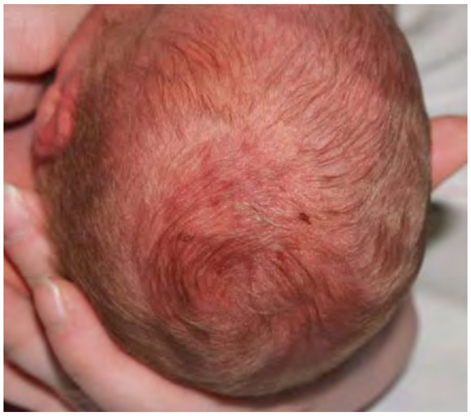

Face

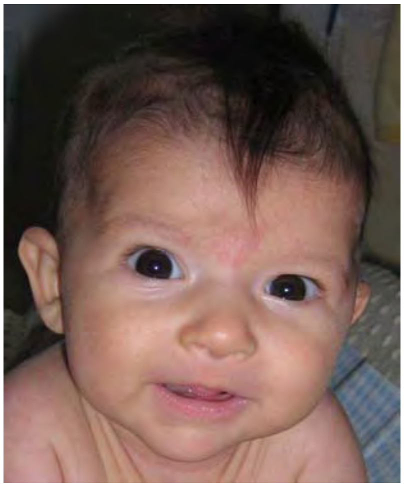

Look at the baby's face and notice if it appears normal. If the face looks strange and you are unsure if the baby is normal or not, make a note on the form so that the baby can be checked by the doctor or examiner. When examining the face, you will check the eyes, ears, nose, and mouth.

Chin

Normal chin

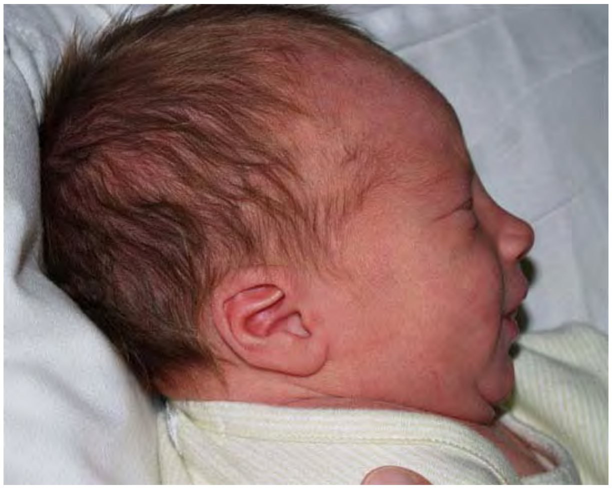

Micrognathia (small chin)

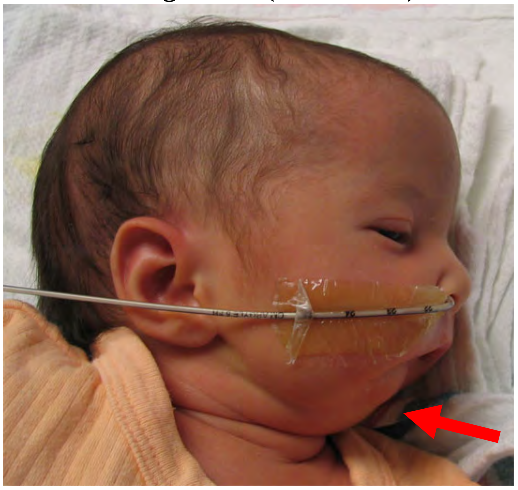

Mouth and palate

Check to see if the palate and mouth are normal. Look for the presence of cleft lip. To check the palate, use a clean finger (preferably with a glove on it) to feel the inside of the mouth for a cleft palate.

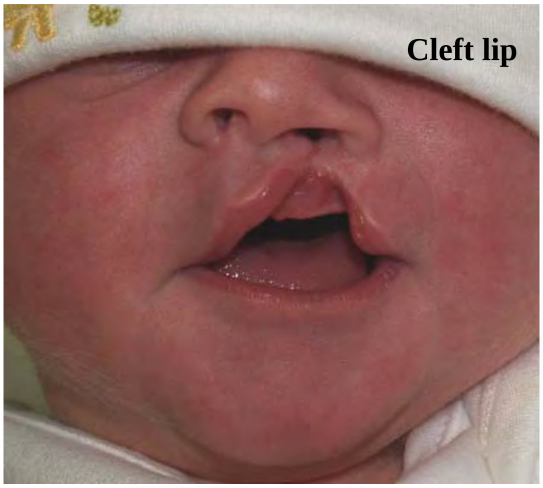

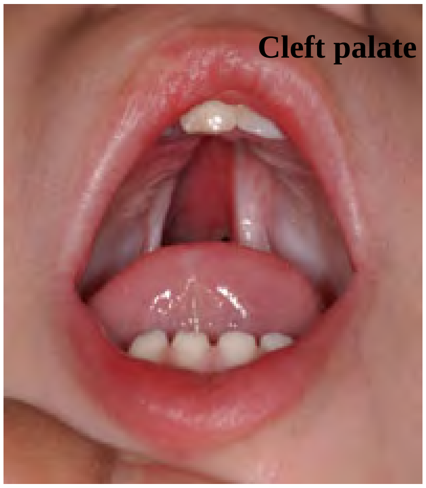

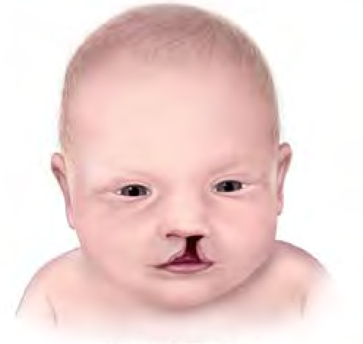
Baby with cleft lip

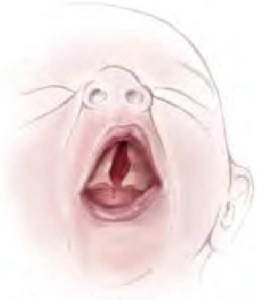
Baby with cleft palate

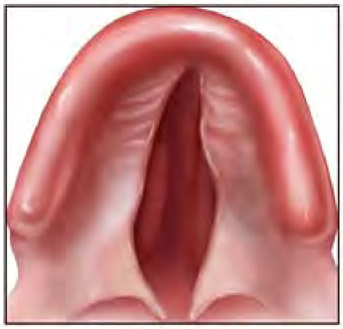
Cleft palate

Lip and philtrum

A smooth philtrum can be associated with certain disorders and should be noted. When examining the philtrum, the infants face needs to be relaxed, the face should not be crying or smiling.

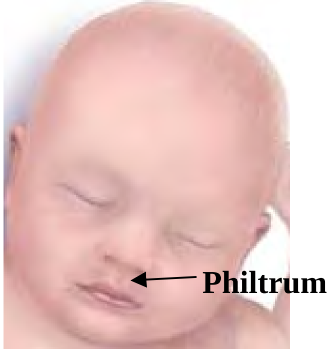

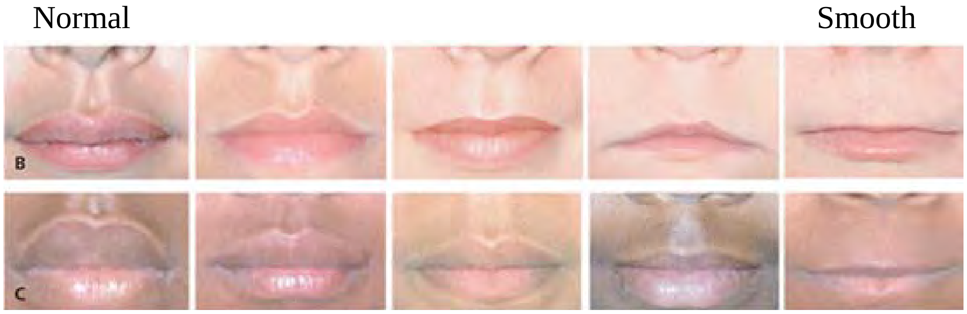

Dr. Susan Astley PhD

Nose

Verify that the nose has 2 nostrils and both nostrils are open. **Choanal atresia** (a blocked nostril) is checked for by seeing if the infant can breathe through both nostrils. To check this, one should gently place a finger over ONE nostril to occlude (block) it. When the left nostril is closed, you are checking that the right side is open and the baby is able to breath. Once you have blocked the left nostril, you should then block the right nostril. When the right nostril is blocked, you are checking that the baby can breathe through the left nostril. Infants do not know how to breathe through their mouths, so if the nose is obstructed, they will have difficulty breathing.

Ears

All abnormalities of the ear, including an ear tag, ear pit, abnormally shaped ear, or too small ear should be recorded on the form, and a picture should be taken to document the abnormality.

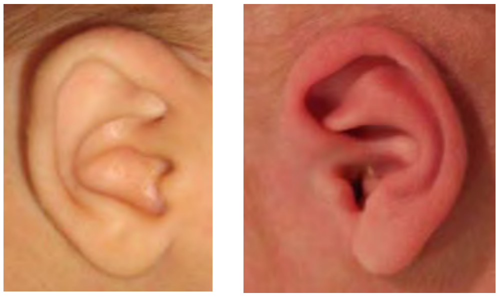

Normal

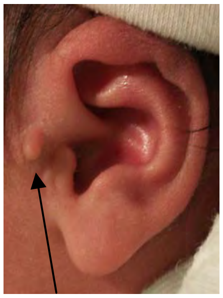

Ear tag

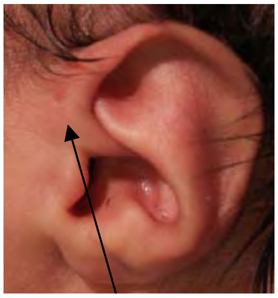

Ear pit

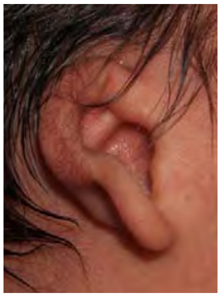

Microtia
(ear too small)

Eyes

The eyes should be clear. Newborn babies are sensitive to the light and therefore they tend to keep their eyes closed. If you shade the eyes with your hands, sometimes this creates enough protection from the light that the baby will open their eyes naturally and you can then examine them. If the eyes are cloudy, this suggests ccongenital cataracts, as shown in the picture.

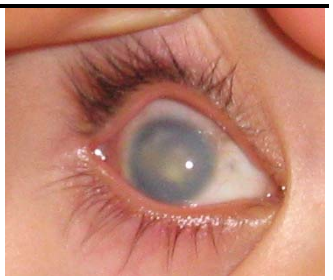

Cataract

Chest

Look at the shape, symmetry, and location of the nipples. Also note if there are any accessory nipples.

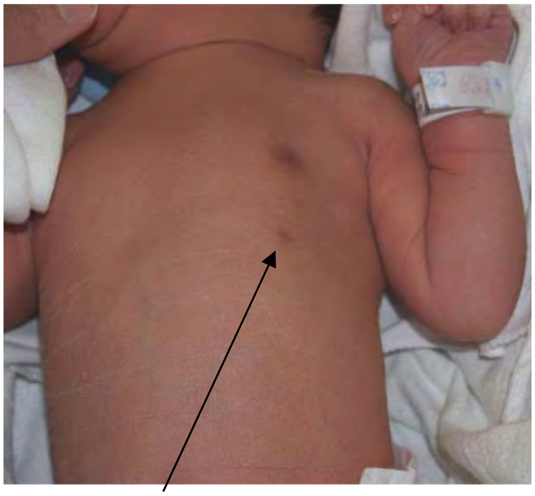

Extra nipple (not significant and not necessary to report)

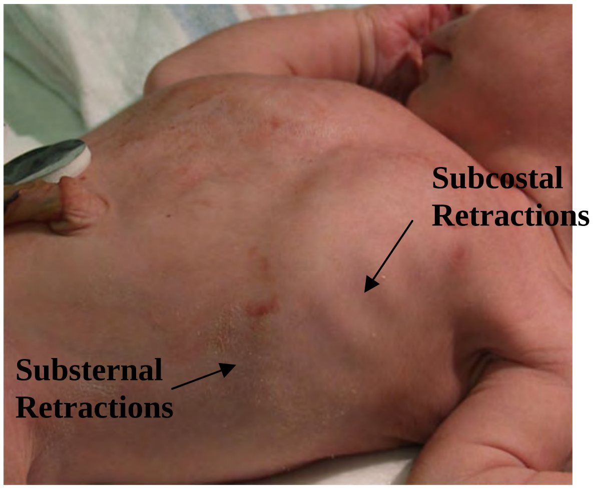

Chest Indrawing (Retractions)

While observing the chest, make a point to check if any of the following are present:

- Chest indrawing
- Nasal flaring
- Tracheal tug
- Head bobbing

These are signs of respiratory distress. If you notice a baby has any of these, ask for help! These symptoms should be recorded on the form under chest.

Abdomen

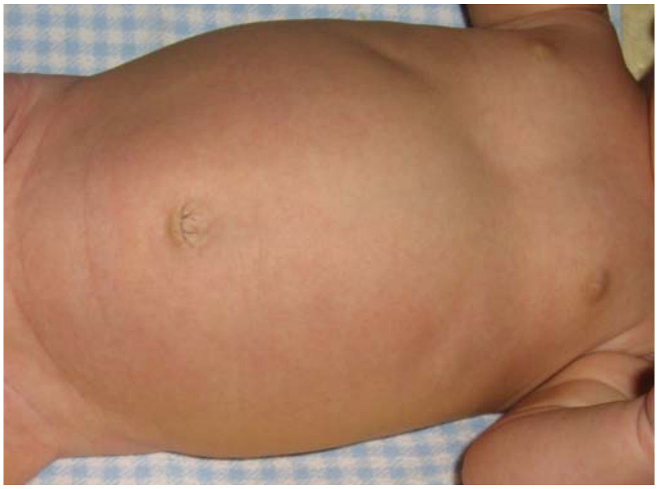

Normal Abdomen

When conducting the abdominal examination, observe if the abdomen appears distended. If you believe the abdomen is distended, contact a medical doctor for further examination.

You should palpate (press on) the abdomen to make sure it is soft. If it is hard, or if you feel a mass, you should ask a doctor to check the baby.

Diastasis Recti (also known as abdominal separation) is a disorder defined as a separation of the rectus abdominis muscle into right and left halves. You will see a bulge in the middle of the abdomen as shown in the picture. This is a NORMAL finding and usually resolves over time and does not need to be recorded.

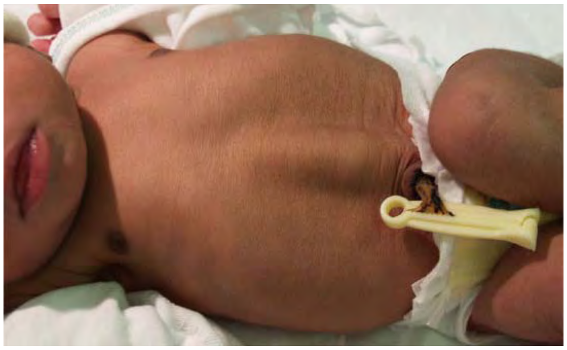

Umbilicus

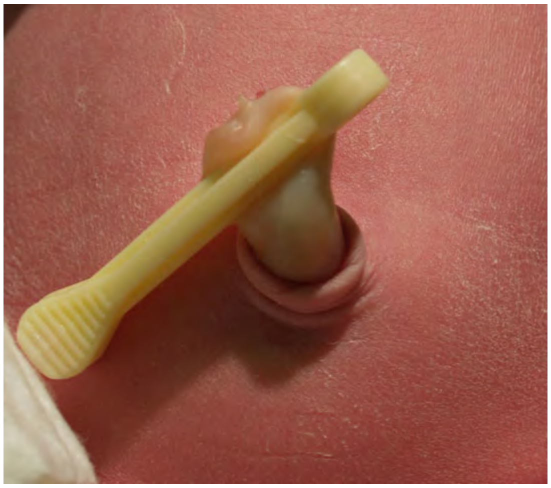

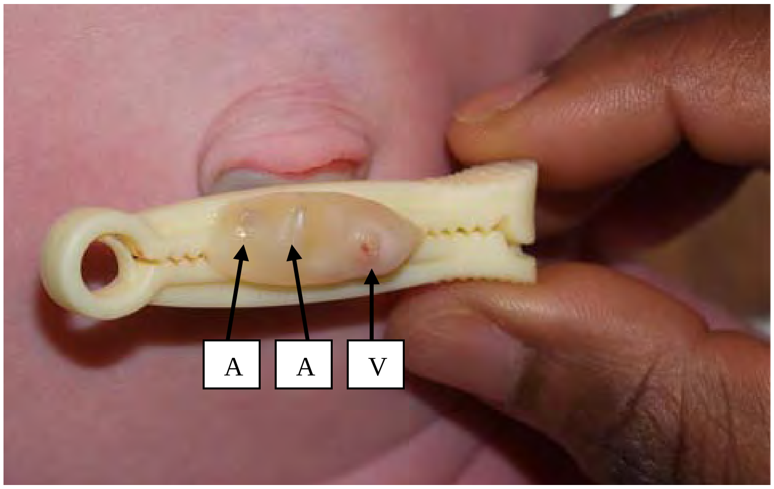

Careful inspection of the umbilicus should include checking the blood vessels. The umbilical cord usually has 3 blood vessels - 2 arteries (A) and 1 vein (V). The vein is the largest of the 3 vessels. Check if all 3 vessels are present. If only two are found, the baby is at risk of having other more serious congenital abnormalities and this should be noted on the form. If the baby is more than a few hours old, you may not be able to see any of the vessels.

Umbilical hernia

This is a defect in the muscles around the umbilicus, which can allow fluid and intestinal contents to bulge out. When you push on the bulge, it should return easily into the abdominal cavity (this is called “reducing a hernina”). If you cannot reduce the hernia, tell a doctor. If an umbilical hernia is small, it will resolve on its own. All umbilical hernias should be recorded on the form under ‘Abdomen’.

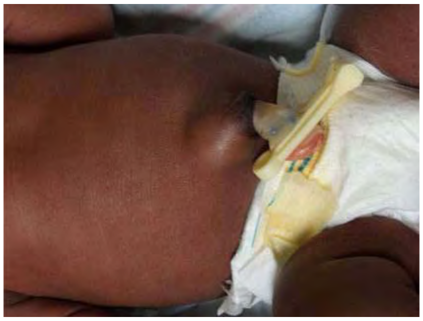

Abdominal Malformations

It is possible that a baby can be born with the internal organs on the outside the abdomen. There are 2 different types of malformations in which this occurs.

An **OMPHALOCELE** occurs when the internal organs protrude through the umbilicus. In this case, they are generally enclosed in a sac, although sometimes this sac will break during the birth process.

In **GASTROSCHESIS,** the malformation is slightly off to the side, and the organs are not protruding through the umbilical cord. In gastroschesis, the internal organs are NOT enclosed in a sac.

If either an omphalocele or gastroschisis are present, notify a clinician!!

Omphalocele

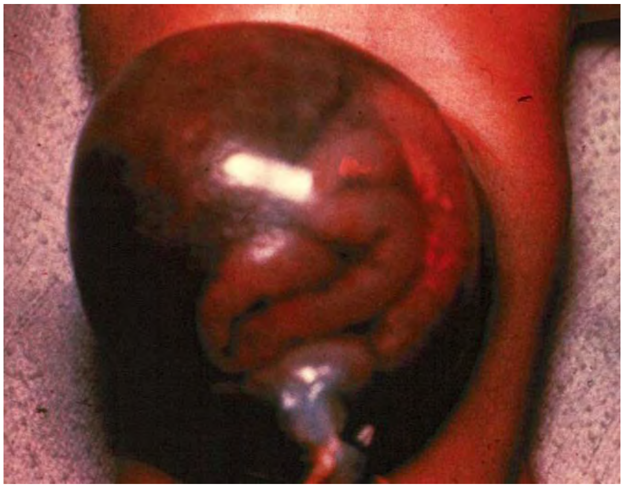

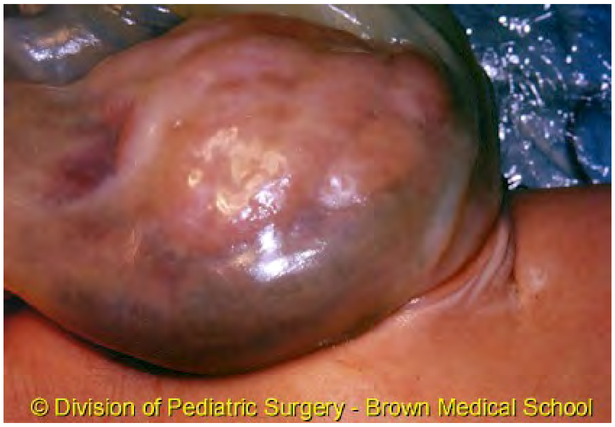

Gastroschesis

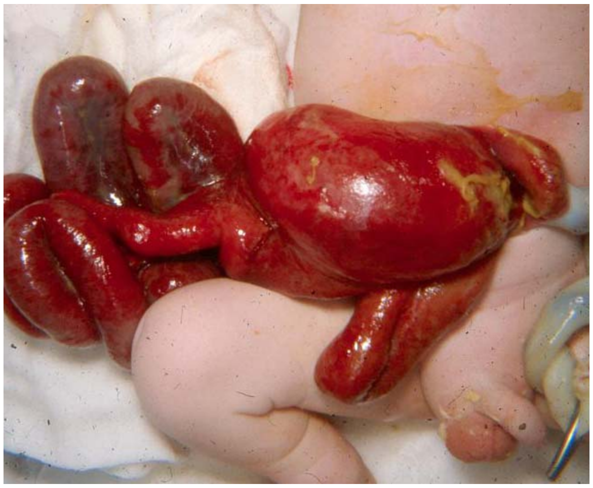

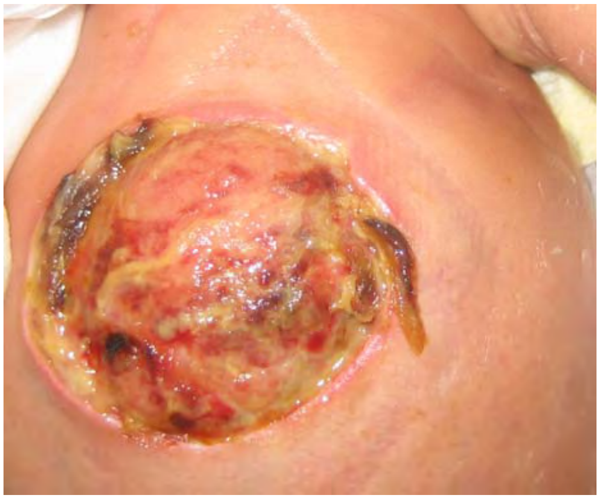

Below is a drawing of an infant with an **omphalocele**. Notice how the infant's intestine and other abdominal organs stick out of the belly button into the umbilical cord.

Omphalocele

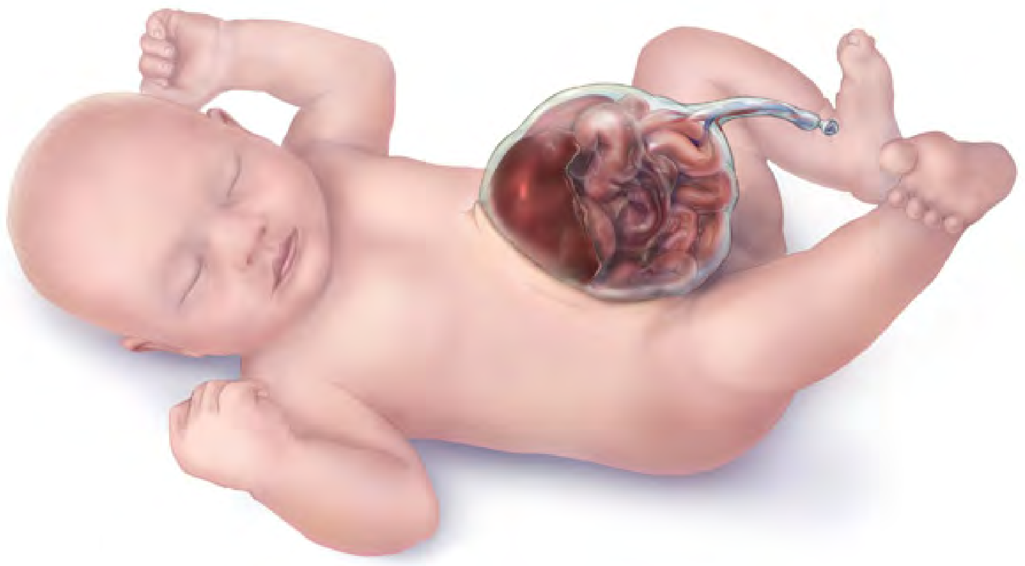

Below is a drawing of an infant with **Gastroschisis**. Notice how the infant's intestines protrude out of the body through a small hole in the body wall beside the umbilical cord. The body wall defect can be small or large and other organs such as the liver can also be involved.

Gastroschesis

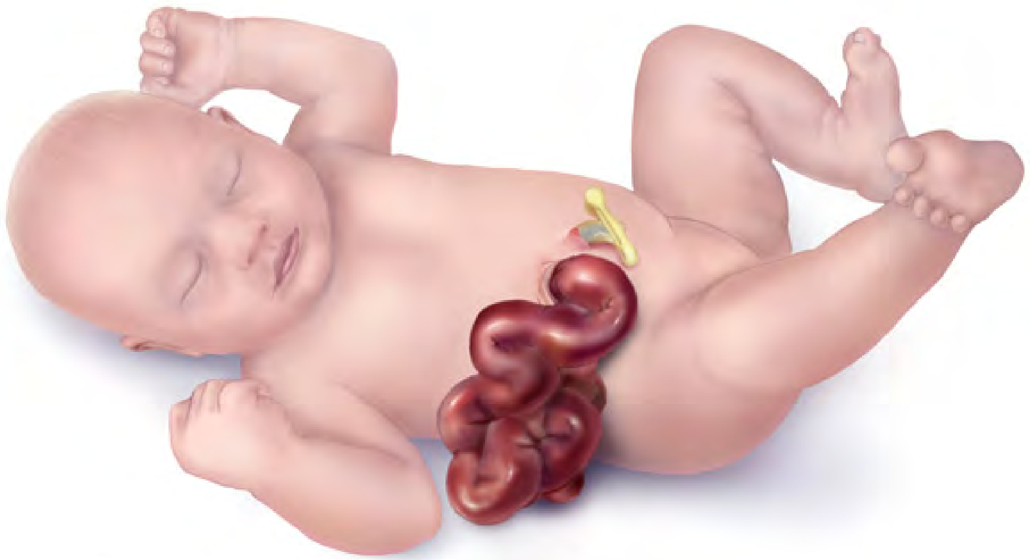

Limbs

Observe the arms, legs, hands, feet, fingers and toes. Make sure there are 5 fingers and 5 toes. If you find any abnormalities, describe them on the form. Don't forget to take a picture.

Normal Limbs

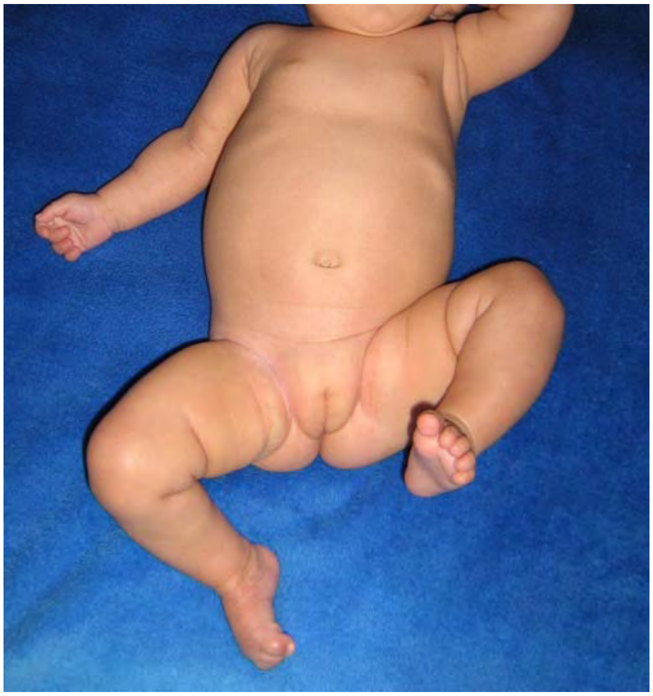

Fingers

Syndactyly of fingers or toes mean that 2 digits are fused together, while polydactyly is the word used when there are more than 5 fingers or toes on one extremity.
Some babies may have a small "extra" finger dangling off from the side of the little finger. This is often attached by only a small piece of skin, and will usually fall off. A string can be tied tightly to speed the process of this falling off. This condition can be seen on the feet as well.

Post axial polydactyly (extra "dangling" finger)

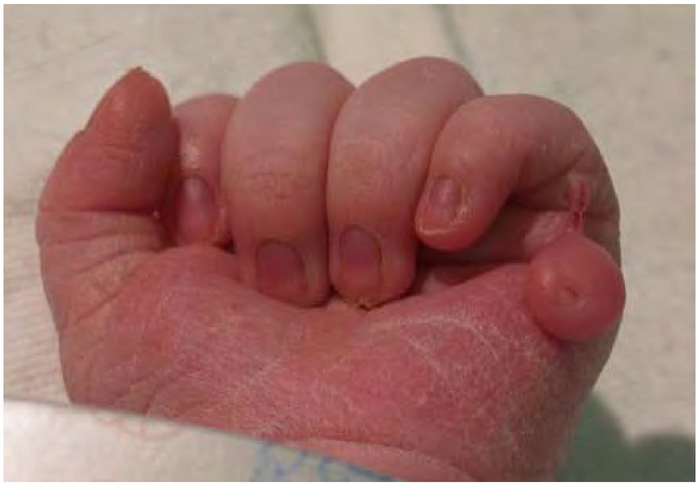

Syndactyly (fused fingers)

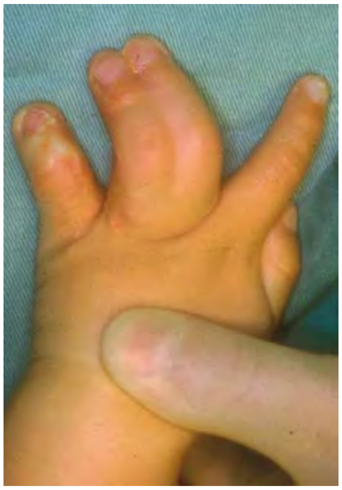

Toes

Post axial polydactyly

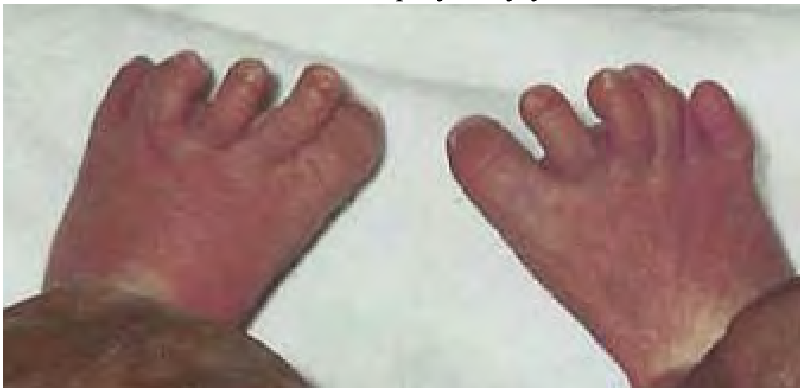

Syndactyly (fused toes)

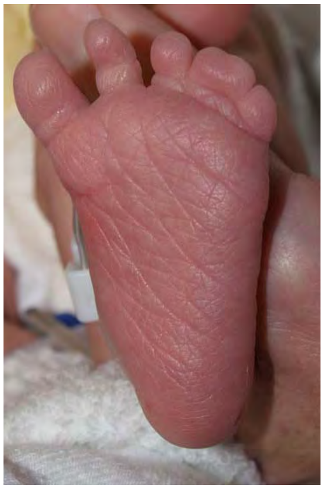

Club foot

Club foot describes an abnormal in-turning of the feet. Many babies will hold their feet slightly turned in, however, you should easily be able to turn the feet so they are straight. If you cannot easily do this, it suggests club foot.

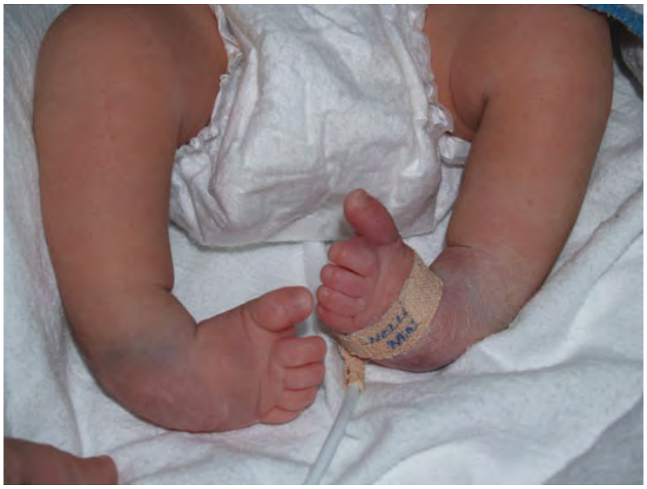

Genital exam - Male

Check to see if the genitalia appear normal. Verify that both of the testes have descended. You should be able to feel the testes within the scrotum- in babies they are usually about 1 cm in size or a bit smaller. In babies, the testes will sometimes be drawn up into the groin (into the inguinal canal), but you should be able to gently bring the teste into the scrotal sac.

Normal male genitalia

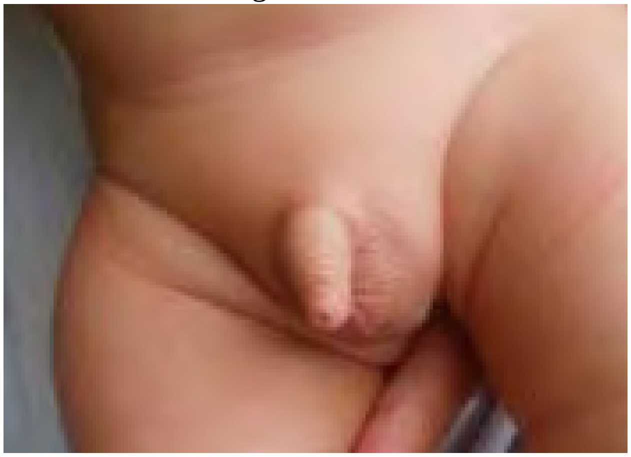

Hypospadias

Hypospadias is an abnormal condition in boys in which the urethra (the opening where urine comes out) opens on the underside rather than at the tip of the penis. A 'hooded foreskin' strongly suggests the presence of hydrospadias. In the photo below, you can see how the penis opens on the underside of the shaft.

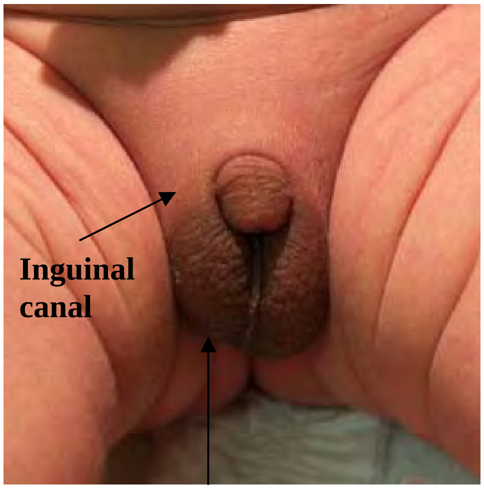

Scrotal sac

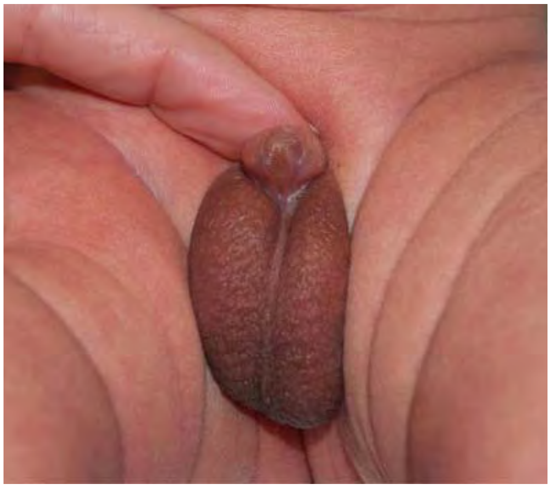

Swollen Scrotal Sac
Some newborn boys will have very large scrotal sacs. This suggests the presence of either a **hydrocele** or an **inguinal hernia**.

A **hydrocele** is fluid-filled sac surrounding the testicle, which is generally not harmful and not painful. Although enlarged, the hydrocele should feel soft and fluid filled, and will not be painful or red. When you palpate the scrotum, you should notice that the testicles are present within the fluid filled sac, although they will be small (about the size of a pea). If you press on the scrotum, you can generally not reduce the size of the scrotal swelling.

An **inguinal hernia** occurs when there is a connection between the abdomen and the scrotum, which can allow intestinal contents to enter the scrotum. In general, you can push the abdominal contents back into the abdomen by applying gentle pressure to the sac (reducing the hernia). Once you let go, however, the sac will usually refill. This should not cause the child pain. Sometimes, the bowels can get stuck inside the scrotal sac. If this occurs, you may not be easily able to push the contents back into the abdominal cavity. If the bowels become stuck inside the scrotal sac, the blood supply to the intestine may be decreased, which can result in death of that part of the intestine. If part of the intestines die, this may be painful. This is very serious and may be life threatening.

A hydrocele generally feels softer than a hernia. In order to differentiate the two, you can shine a light onto the scrotal sac. Place the light on the underside of the scrotal sac. If the light shines through, as in the picture, this is more suggestive of a hydrocele.

Hydrocele

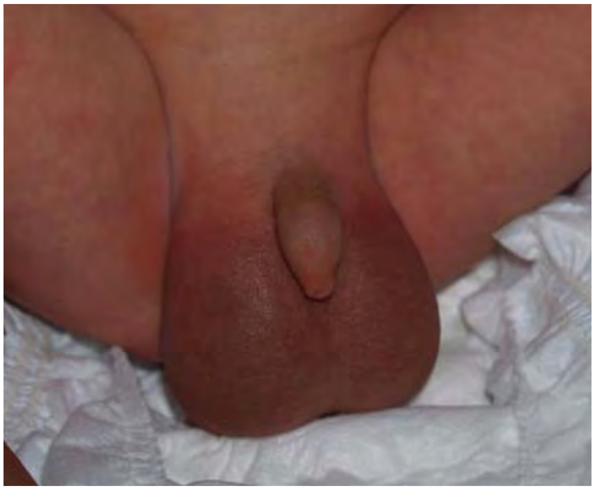

llumination of a hydrocele- note that the light is seen easily through the scrotal sac

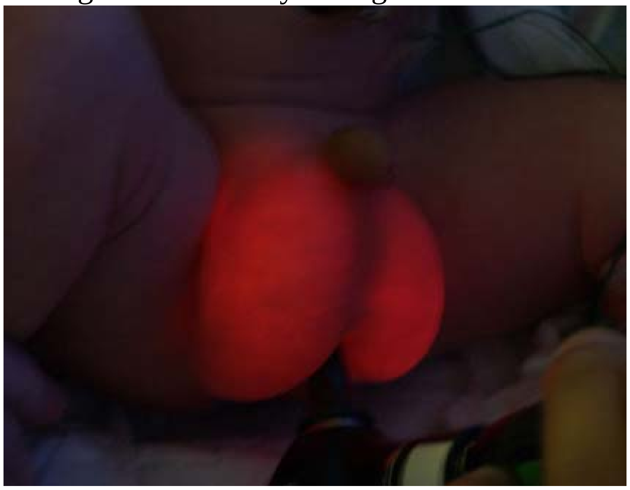

Genital Exam – Female

Female infants may have some whitish discharge or even some small amounts of blood from the vagina - this is normal. When examining the female genitalia, try to separate the labia as shown in the photo on the right. This will allow you to identify if the labia are fused (stuck together).

Normal Female Genitalia

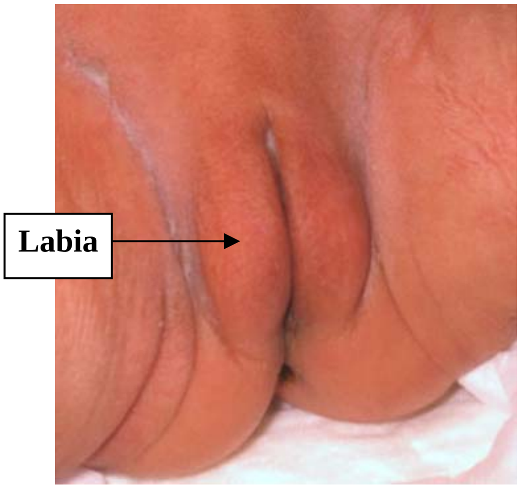

Normal Female Genitalia

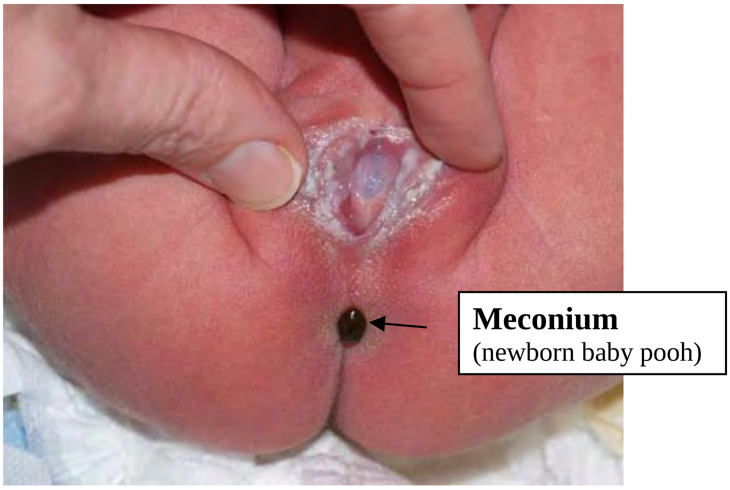

Anus

An Imperforate Anus is a congenital defect of the anus; there is partial or complete obstruction of the anal opening. During the exam, you should verify that the baby's anus is patent. This can be done by taking a rectal temperature and inserting the thermometer 1-2 cm into the rectum or by inserting a gloved finger 1- 2 cm into the rectum in order to confirm that the anus is patent. Note in the photo on the right, there is no opening for the anus! An imperforate anus can be excluded by careful routine examination of the area. Any unusual appearance warrants careful investigation. A baby with imperforate anus may still pass meconium if there is another associated abnormality, such as an abnormal connection between the vagina and rectum (recto-vaginal fistula). Therefore, even if you see meconium, make sure to still verify the presence of an anal opening.

Normal Anus

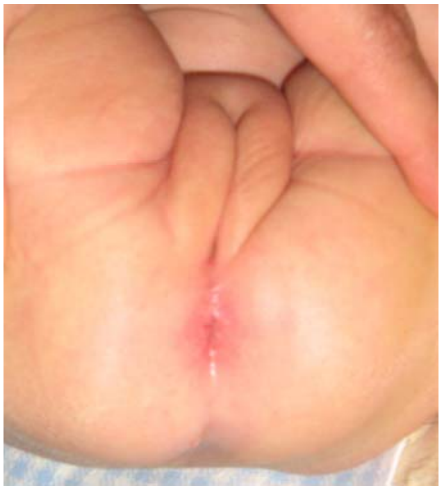

Imperforate Anus

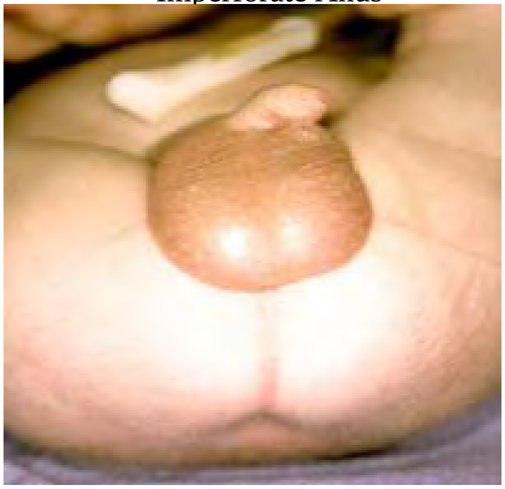

Back & Spine

Normal Back

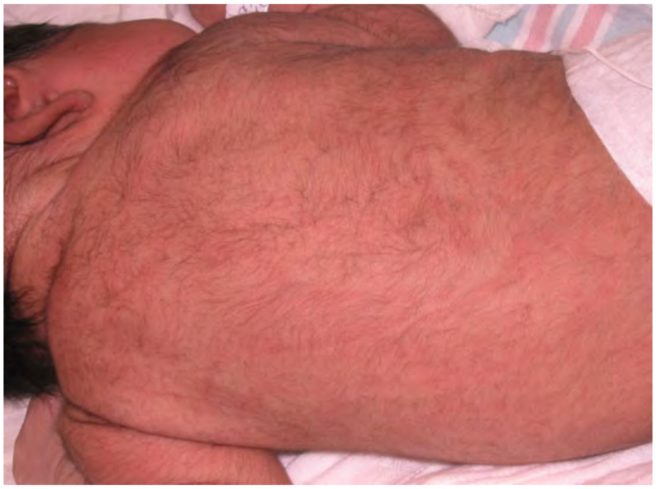

Look at the baby's back. Pay careful attention to any abnormalities which might be located along the midline or along the spine. Look for birthmarks, dimples, or tufts of hair along the spine. These may be a sign of a more serious problem such as Spina Bifida. Examine the spine to make sure there are no abnormalities in the bones. Any abnormalities that you find along the midline of the back should be recorded, including any dimples or pits, and skin tags, any tufts of hair, or anything else which does not look normal. You should also take a picture to document the abnormality.

Sacral Skin Tag

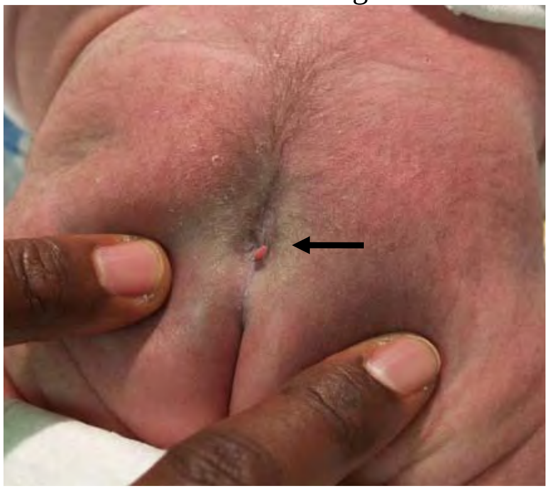

Sacral Dimple

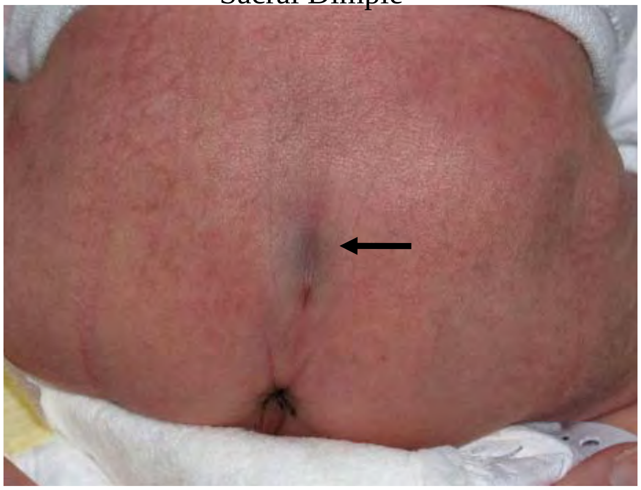

Saccrococcygeal Teratoma

A teratoma is a germ cell tumor found in the midline of the body. In newborns, the most common location where these tumors occur is at the sacrococcygeal region- at the base of the spine. This is a mass of tissue which protrudes from the back, and does not come out of the spinal cord as in Spina Bifida.

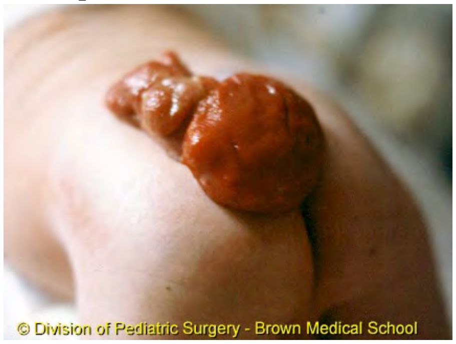

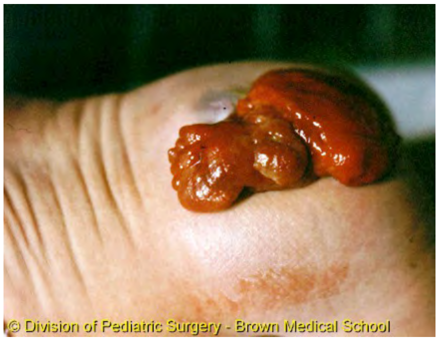

Neural Tube Defects

The neural tube is a narrow channel that folds and closes during the 3rd and 4th weeks of pregnancy to form the brain and spinal cord. Incomplete closure of this tube results in several different birth defects:

- Anencephaly
- Encephlomyelocele
- Spina Bifida.

Anencephaly – this birth defect is characterized by a baby missing parts of the brain, skull, and scalp. Babies with this condition often are born without the thinking part of the brain. The remaining brain tissue is often exposed, meaning, it is not covered by bone or skin.

Encephalocele is a sac-like protrusion of the brain through an opening in the midline of the skull.

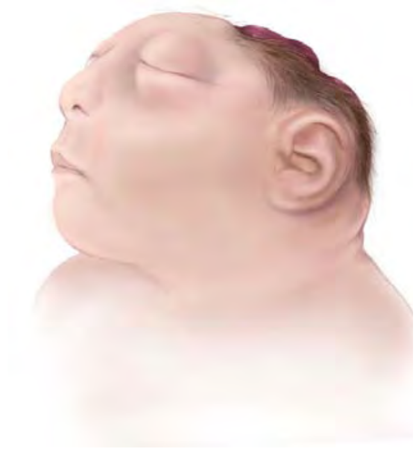

Anencephaly

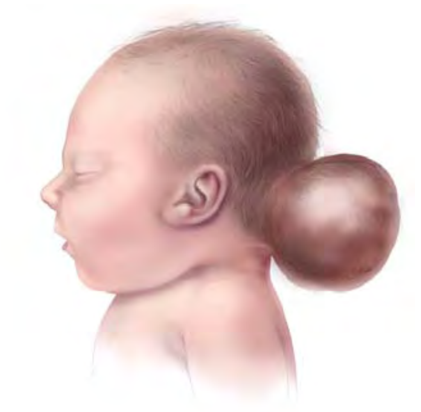

Encephalocele

Spina Bifida

Spina Bifida is a malformation where the bones around the spinal cord do not close all the way. Sometimes, the skin is open as well, and the spinal cord is exposed.
If you see this, tell a clinician!

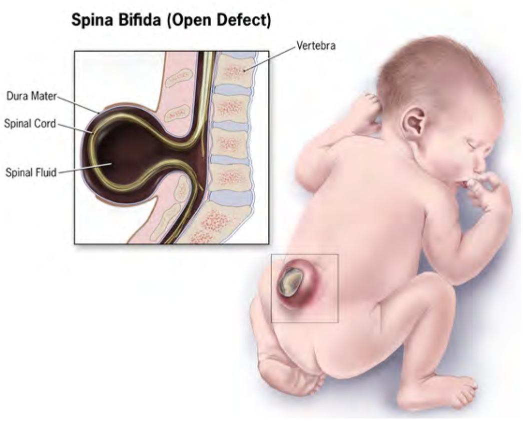

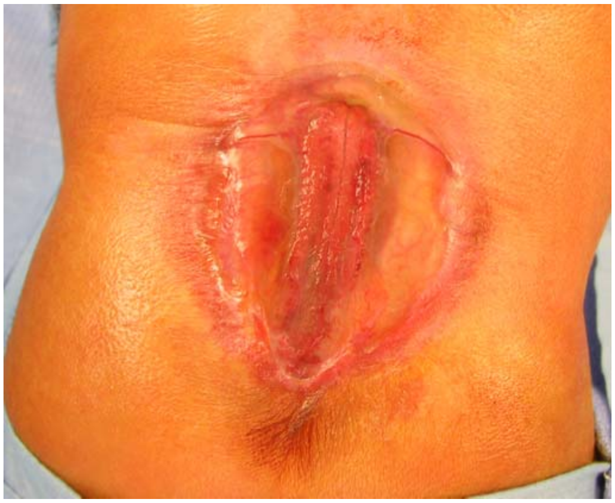

Skin

There are many different types of rashes commonly seen in newborns. Rashes do not need to be recorded on the examination form! However, since you are examining the baby, the parents may ask you about the rashes. Rashes may be described with words such as: Red, Raised, Flat, or Pustules. Below are photos of 4 normal rashes that you may see.

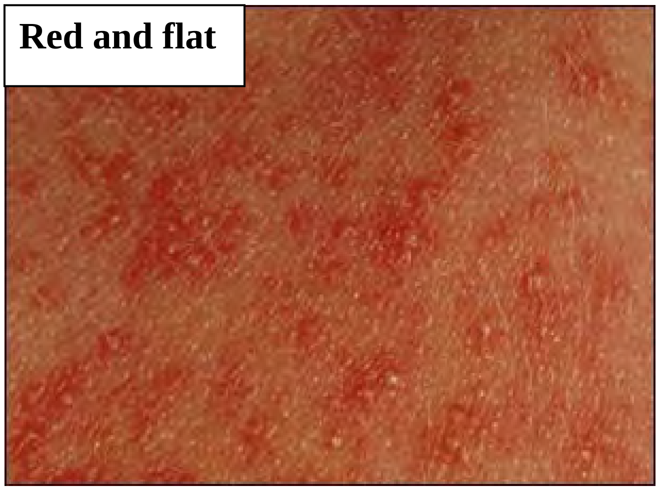
Red and flat

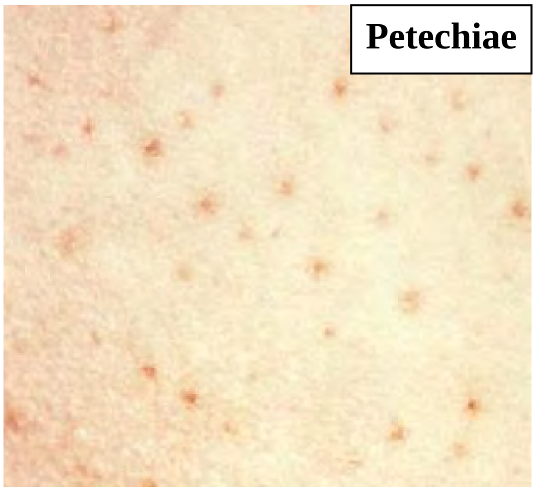
Petechiae

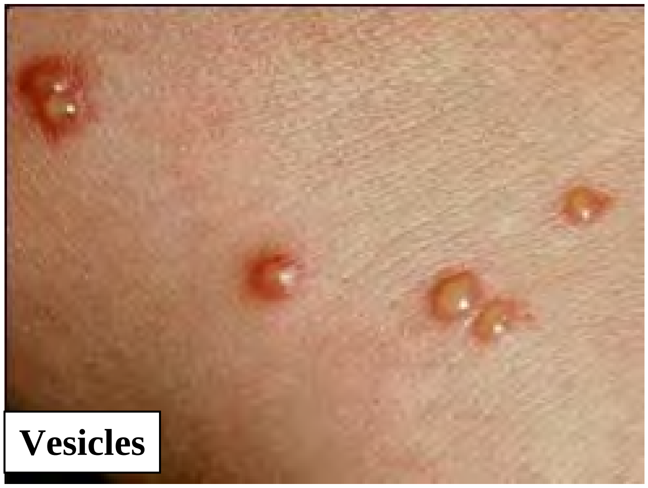
Vesicles

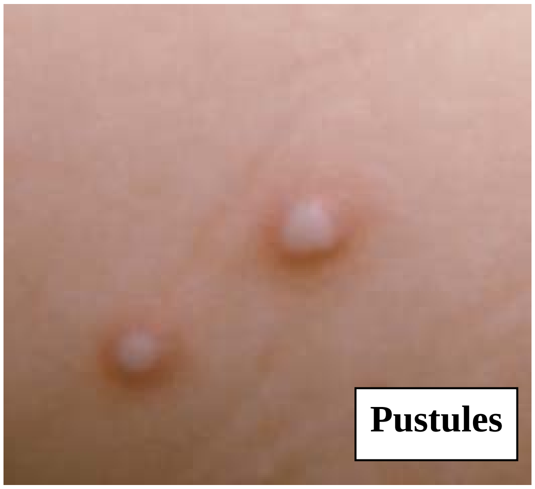
Pustules

Normal Newborn Rashes

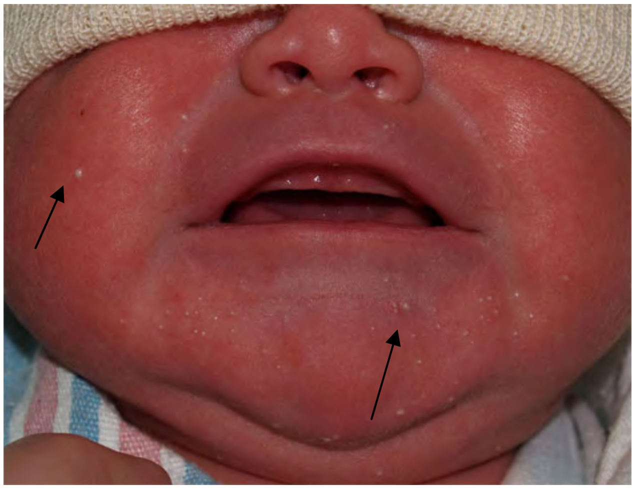

Milia - are tiny white bumps or. small cysts on the skin

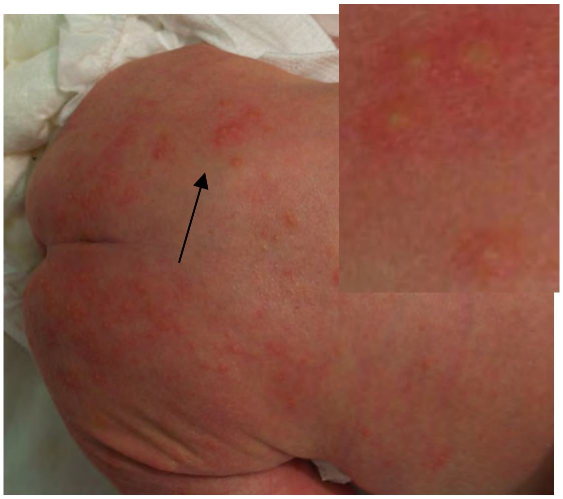

Erythema Toxicum

Birthmarks

Birthmarks do not need to be recorded on the examination form! But like rashes, parents may ask you about it. Mongolian spots are blue marks which are commonly found on the buttocks. A red mark on the back of the neck, or above the eyes is referred to as a stork bite. Both of these birthmarks are fairly common and are not dangerous.

Mongolian Spots

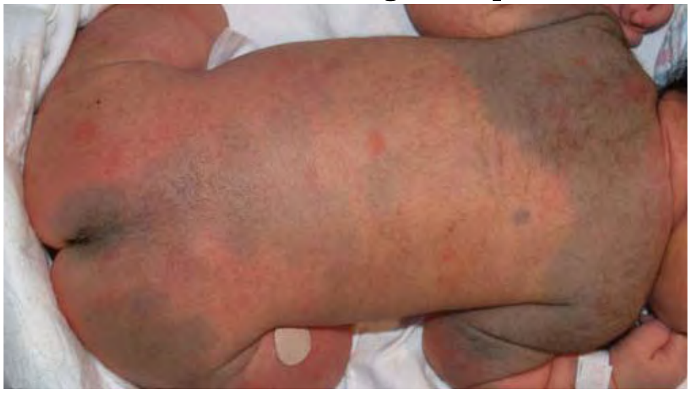

Stork bite

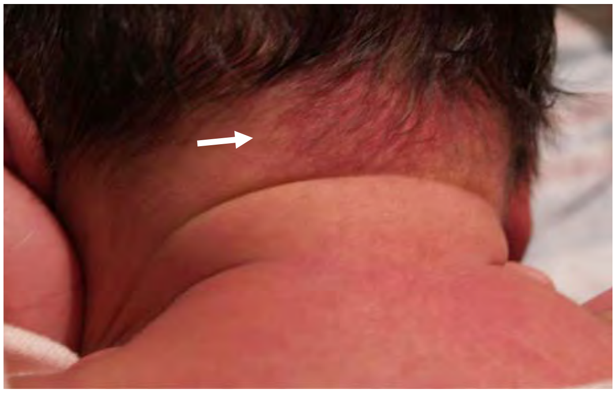

Neurological Examination

As part of the newborn assessment, we'll also be conducting a neurological examination. This means we'll be looking at the muscles and nerves. We want to note if the infant appears to have normal muscle tone, or, if the infant appears too floppy or too stiff. We'll also be observing if the infant is moving all of the limbs normally. If anything appears strange, please describe your findings.

Hypotonia (decreased muscle tone)

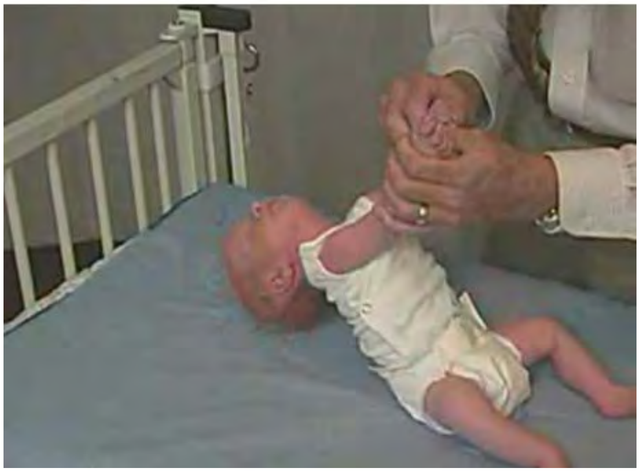

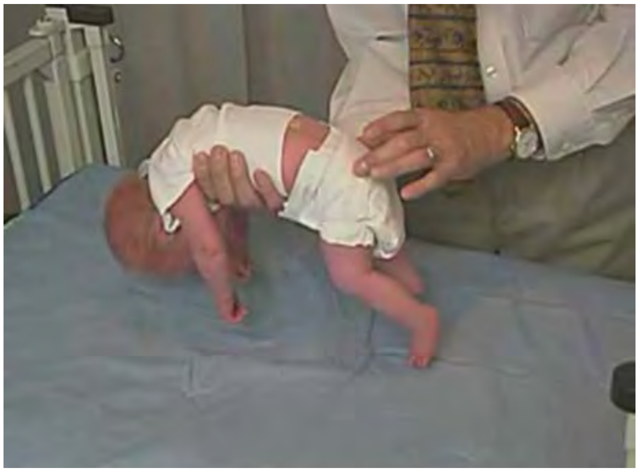

Photos: http://library.med.utah.edu/pedineurologicexam/

III. SUMMARY

In conclusion, never be worried to ask a doctor or medic to see child if you suspect the child has an abnormality. If you are unsure if an abnormality is present, or if you suspect one, a doctor must be notified. In addition, please take a picture of any suspected abnormalities and document them on the form.

A routine physical examination takes only a few minutes and should be carried out on ALL infants as soon as possible after birth; all babies should have an exam in the first week of life. Many of the serious correctable congenital malformations can be detected at birth or within a few days. Early diagnosis of certain abnormalities greatly increases the chances of survival and can greatly reduce permanent residual disability.

IV. TRAINING

Prior to performing newborn examinations alone, all staff should witness at least 3 examinations performed by a trained examiner, and then should be observed doing 10 examinations and checked off to make sure that they have mastered the examination. Any staff who examines fewer than 4 infants in a month should be re-checked every 2 months.

V. TRAINING LOG

Staff member name: ____

Observation of a trained examiner **Signature of the examiner**

Observation 1: Date: |__|__|/|__|__|/|__|__| dd/mm/yy ______________________________

Observation 2: Date: |__|__|/|__|__|/|__|__| dd/mm/yy ______________________________

Observation 3: Date: |__|__|/|__|__|/|__|__| dd/mm/yy ______________________________

Examination with a trained observer **Signature of the examiner**

Examination 1: Date: |__|__|/|__|__|/|__|__| dd/mm/yy ______________________________

Examination 2: Date: |__|__|/|__|__|/|__|__| dd/mm/yy ______________________________

Examination 3: Date: |__|__|/|__|__|/|__|__| dd/mm/yy ______________________________

Examination 4: Date: |__|__|/|__|__|/|__|__| dd/mm/yy ______________________________

Examination 5: Date: |__|__|/|__|__|/|__|__| dd/mm/yy ______________________________

Examination 6: Date: |__|__|/|__|__|/|__|__| dd/mm/yy ______________________________

Examination 7: Date: |__|__|/|__|__|/|__|__| dd/mm/yy ______________________________

Examination 8: Date: |__|__|/|__|__|/|__|__| dd/mm/yy ______________________________

Examination 9: Date: |__|__|/|__|__|/|__|__| dd/mm/yy ______________________________

Examination 10: Date: |__|__|/|__|__|/|__|__| dd/mm/yy ______________________________

Approved to do examinations alone once 3 observations and 10 examinations have been completed.

Re-check 1: Date: |__|__|/|__|__|/|__|__| dd/mm/yy ______________________________

Re-check 2: Date: |__|__|/|__|__|/|__|__| dd/mm/yy ______________________________

Re-check 3: Date: |__|__|/|__|__|/|__|__| dd/mm/yy ______________________________

VI. APPENDIX

A

Abdomen 16
- Diastasis Recti (Abdominal separation)....17
- Umbilical hernia 17

Abdominal wall defects....18
- Gastroschesis 18
- Omphalocele....18

Acrocyanosis ....*See* Color
Anencephaly....26
Anterior fontanelle....10
Anus....24

B

Back & Spine....25
- Saccrococcygeal teratoma....25
- Sacral dimple 25
- Sacral skin tag....25
- Spina Bifida 25

Belly button (Umbilicus)....17
Birthmarks....28
Blue color ....*See* Color
Blue feet/hands ....*See* Color
Blue lips....*See* Color
Bruising 13
Bulging fontanelle ....10, See Anterior fontanelle

C

Caput 12
Cataracts, congenital....15
Cephalohematoma ....*See*
Chest....16
Chest indrawing....*See* Chest
Chin 13
- Micrognathia (Small chin)....13
- Normal....13

Choanal atresia (Blocked nostril) ....*See* Nose
Cleft lip....14
Cleft palate....14
Club foot....21
Color....9
Congenital malformations
- causes....5

Cyanosis (Blue color)....*See* Color

D

Diastasis Recti (Abdominal separation) 17

E

Ears....15
- Ear pit 15
- Ear tag....15
- Microtia (Small ear)....15
- Normal....15

Encephalocele....26
Encephlomyelocele....26
Enlarged head ....*See* Hydrocephalus
Erythema toxicum....28
Exam logistics
- Length of the exam 6
- Materials needed....7
- Order of the exam 8
- Where to conduct....7

Eyes 15
- Congenital cataracts....15

F

Face 13
Fingers....20
- Polydactyly (Extra finger....20
- Post axial polydactyly....20
- Syndactyly (Fused fingers) 20

Fontanelle 10

G

Gastroschesis....19
Gastroschesis (Abdominal wall defect)....18
Genital exam....22
- Female 24
- Male 22

H

Head and skull bones
- Caput....12
- Fontanelle 10
- Molding and Caput 11
- Normal molding....11

Head bobbing....*See* Chest
Hydrocele (Testicles)....23
Hydrocephalus (Enlarge head) 12
Hypospadias (Penis) 22
Hypotonia 29

I

Imperforate anus....24
Inguinal hernia....23
Introduction 4

J

Jaundice (Yellow skin) ....*See* Color

L

Limbs....20
Lip and philtrum....14

M

Measurements....9
Head circumference....9
Length....10
Respiratory rate....9
Meconium.... *See* Anus
Milia (Rash)....28
Molding....11
Mongolian spots.... *See* Birthmarks
Mouth and palate....14

N

Nasal flaring.... *See* Chest
Neural tube defects....26
Anencephaly....26
Encephlomyelocele....26
Spina Bifida....26
Neurological examination....29
Hypotonia....29
Nose....15
Blocked nostril....15

O

Omphalocele....19
Omphalocele (Abdominal wall defect)....18

P

Pale.... *See* Color
Perioral cyanosis (Blue around the mouth).. *See* Color
Polydactyly (Extra finger)....20

R

Rashes....27
Flat....27
Pustules....27
Raised....27
Red....27

S

Saccrococcygeal teratoma....25
Sacral dimple....25
Sacral skin tag....25
Scrotal sac....23
Skull bones....11
Spina Bifida....25, 26
Stork bite.... *See* Birthmarks
Summary....29
Sunken fontanelle....10, *See* Anterior fontanelle
Syndactyly (Fused fingers)....20

T

Toes....21
Post axial polydactyly....21
Syndactyly (Fused toes)....21
Tracheal tug.... *See* Chest
Training....29
Training log....30

U

Umbilical hernia....17
Umbilicus (Belly button)....17

Y

Yellow skin color.... *See* Color
