# Supplementary material for: Assessment of the safety of antimalarial drug use during early pregnancy (ASAP): protocol for a multicenter prospective cohort study in Burkina Faso, Kenya and Mozambique
Source: Reprod Health. 2015 Dec 4;12:112. doi: 10.1186/s12978-015-0101-0 (PMC4670540; doi:10.1186/s12978-015-0101-0)
Supplement: Additional file 2: — Ultrasound Reference Manual for Pregnancy Dating. (PDF 3017 kb) [file 12978_2015_101_MOESM2_ESM.pdf]

Ultrasound Reference Manual for Pregnancy Dating

Developed for the Assessing the Safety of Antimalarials during early Pregnancy (ASAP) Study through support from the Malaria in Pregnancy Consortium (MiPc)

January 2012

malaria in pregnancy consortium

DEPARTMENT OF GLOBAL HEALTH
UNIVERSITY of WASHINGTON
GLOBAL MEDICINES PROGRAM

Contact Information:
Rebecca Bartlein, MPH
Research Coordinator, Global Medicines Program
Department of Global Health
University of Washington
Harris Hydraulics Building, Room 320A
1705 NE Pacific St.; Box 357965
Seattle, WA 98195-7965
Tel: (206) 543-8355
Fax: (206) 685-8519
Email: bartlein@uw.edu
www.globalmedicines.org

Terms of Use: This manual is intended for use by the ASAP project and may be reproduced and used freely if properly cited. Selected images and text should also be credited to the INTERGROWTH Study as stated below.

With thanks to:
Nicole Goldsmith, BA, RDMS – Clinical Ultrasound Instructor
Department of Radiology, University of Washington

Robert Nathan, MD, MPH – Acting Assistant Professor
Department of Radiology, University of Washington

The Project Management Committee of the Assessing the Safety of Antimalarials during early Pregnancy (ASAP) Study: Feiko Ter Kuile, Tinto Halidou, Esperança Sevene, Stephanie Dellicour, Umberto d'Alessandro, Andy Stergachis and Laura Sangaré.

INTERGROWTH-21st Ultrasound Operations Manual http://www.intergrowth21.org.uk/ for providing text and images.
The INTERGROWTH-21st Ultrasound Operations Manual was prepared as part of the INTERGROWTH-21st Project by the International Fetal and Newborn Growth Consortium. The INTERGROWTH-21st Project was supported by Grant ID# 49038 from the Bill & Melinda Gates Foundation to the University of Oxford.

Table of Contents

Introduction 3

System Setup 3

Examining a new patient 4

Measurements 5

- Crown Rump Length (CRL) 5
- Head measurements 8
- Abdominal measurements 13
- Femur length measurement 18

Interpretation 22

Finishing the exam 22

Introduction

This reference manual is intended to be used by a qualified ultrasound instructor as a resource when conducting training activities with nurses, midwives and other clinicians. This manual is also intended to be kept by the trained clinicians as a reference when ultrasound examinations are being conducted. This manual is not intended to substitute for proper training and hands-on experience, but rather as a supplement to it. This manual was developed with the SonoSite 180plus system, and more information about this ultrasound machine can be found at www.sonosite.com.

System Setup

Connect the transducer to the system by pulling up the latch on the transducer connector and rotating it clockwise until it snaps to a stop (Do not force the latch). Align the transducer connector with the connector on the rear of the system and insert it by pushing the transducer connector into the system connector. Turn the latch counter-clockwise until it snaps to a stop.

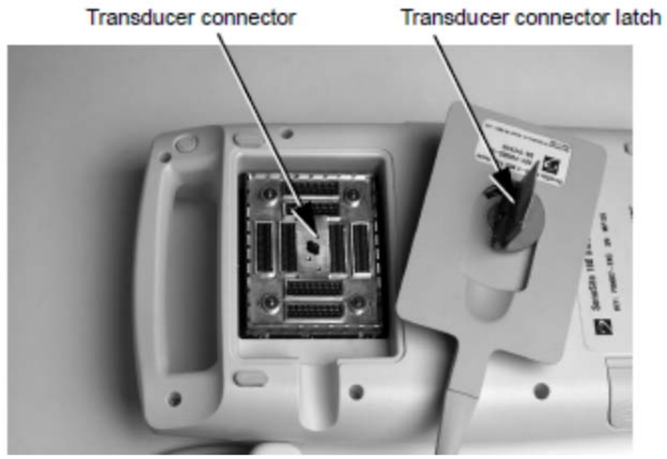

CAUTION: The electrical contacts inside the system transducer connector may be damaged by foreign material or by rough handling. Do not touch the electrical contacts. Keep foreign material out of the connector. Keep transducer connected to the system whenever possible.

1. Plug the system to a wall outlet with a surge protector.

CAUTION: Please use a surge protector as fluctuation in electrical current can ruin the system.

2. Switch the system on by pushing and holding the power switch (see picture below) for about 1 second, until the system beeps.

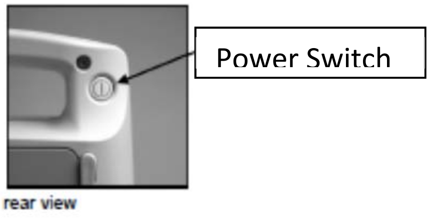

Note: the system goes on standby when there has been no activity; press any system key to wake up the system.

3. Verify the system is connected to power. A power plug icon should appear in the lower right corner of the screen which indicates it is properly connected.
4. Check the time and date is correct by pressing the **Patient** key, selecting system set up from the menu, select date/time and enter the current time (24hr format) and date(year, month, day). Press patient key to return to live image.

Examining a new patient

Preparation of the patient

1. Explain to the patient what you will be doing.
2. The patient should be lying comfortably on her back, the head slightly raised. Apply acoustic coupling agent (gel) liberally to the lower abdomen and put protective tissue around her belt.

Enter patient's detail

1. Press the **Patient** key, from the menu list select exam/patient information, then select new patient.
2. Enter DSS ID under Name* and press **Enter.**
3. Record your code or initials under id and press **Enter.**
4. Enter the ANC number under Accession and press **Enter**.
5. Select OB for exam type and press **Enter**.
6. Enter LMP if known and press **Enter**.
7. Press the **Patient** key to return to the screen for examination.

*DSS ID is recorded under name to preserve anonymity of the photos when transferred for QA. This is because the picture will be saved under "Name".

Obstetric examination

1. Position the probe transversely over the lower abdomen with the orientation mark pointing to the patient's right side.
2. Adjust the gain and focus to produce the best image (important for BPD and HC).
3. Adjust the zoom of the image to improve quality of the image and visibility of the anatomy (important for small fetuses in the 1st trimester).
4. Once you are satisfied with the image quality, start the obstetric exam and measurements.
5. Check for multiple pregnancy (look for number of gestational sacs, embryos or heart beats)
6. Search for the fetal heart beat and confirm fetus viability.

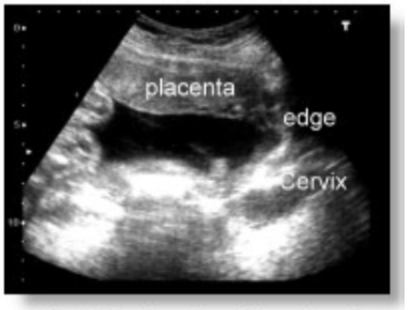

determining the edge of the placenta

Measurements

Heart motion is perceptible from about 6 weeks onwards. The crown-rump length (CRL) is the most reliable parameter for estimating gestational age up to the eleventh week. From the twelfth week onwards, the biparietal diameter (BPD) is more accurate (with HC, AC & FL). For women at the boundary of the 1st trimester (12-15 weeks), do all of the following: CRL, BPD, HC, AC and FL. After the second trimester, precision of gestational age estimation can vary +/- 3 weeks (see interpretation table).

1. Proceed to the measurements in the order that works best with the fetus position.
2. When you identified the body part to measure, freeze the image by pressing the **Freeze** key and then the **Measure** key. A menu will appear on the left-hand side of the screen.
3. On the left hand side menu select the key and select the appropriate measurement using the **Arrow** keys at the bottom of the screen.

Crown Rump Length (CRL)
For women in their 1st trimester (until 15 weeks) measure the crown-rump length (CRL). CRL is measured along the longest axis of the fetus (see image below). Take 3 consecutive measurements and record the average of the 3 measurements. Save all the images using the **Save** key.

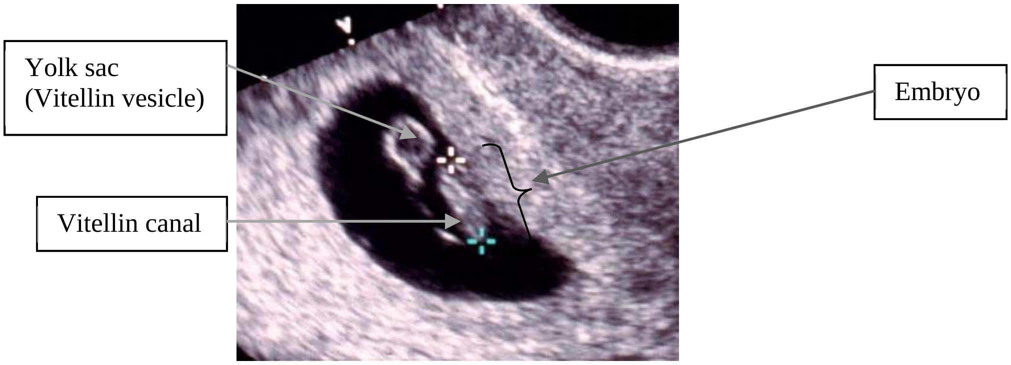

Crown Rump Length Measurements (CRL) before 16 weeks gestation

- ***Gestational age will be calculated from the Last Menstrual Period (LMP).***
  In order to allow accurate assessment of gestation women need to have:
  - A known LMP
  - Regular menstrual cycles with a cycle between 25 and 31 days
  - No hormonal contraception use in the 2 months preceding the LMP
  - No breastfeeding in the 2 months preceding the LMP
  - Spontaneous conception only

- ***This gestation is confirmed by fetal crown-rump length (CRL).***
  The gestational age from the CRL will be calculated automatically by the study ultrasound machine using established charts. If the dating scan is done on another machine, make sure you use the attached chart to look up the estimated gestation.

  When the gestational age based on CRL is within 7 days of that calculated from LMP we will consider the LMP to be reliable, and take it as the true biological date.

Step 1: Identify the fetus

1. Viable?
2. Intrauterine?
3. Singleton?

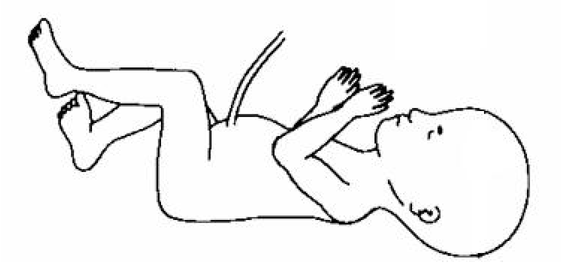

Step 2: Measure the Crown – Rump Length (CRL)

1. Find the mid-sagittal section of the fetus
2. The fetus should be horizontal (at 90º to the angle of insonation)
3. The fetus should be in a neutral position (not hyperextended or flexed)
4. The fetus should fill at least 30% of the image on the monitor.
5. Place the intersection of the calipers on the outer borders of the head and rump.

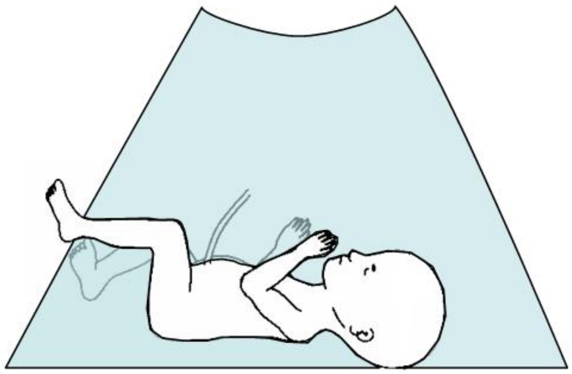

A mid-sagittal section of the fetus, in a horizontal position (90° to the angle of insonation), in a neutral position.

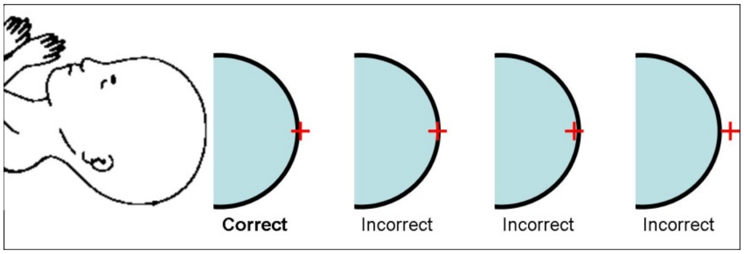

The intersection of the calipers should be placed on the ***outer*** *borders of the head and rump.*

COMMON ERRORS:

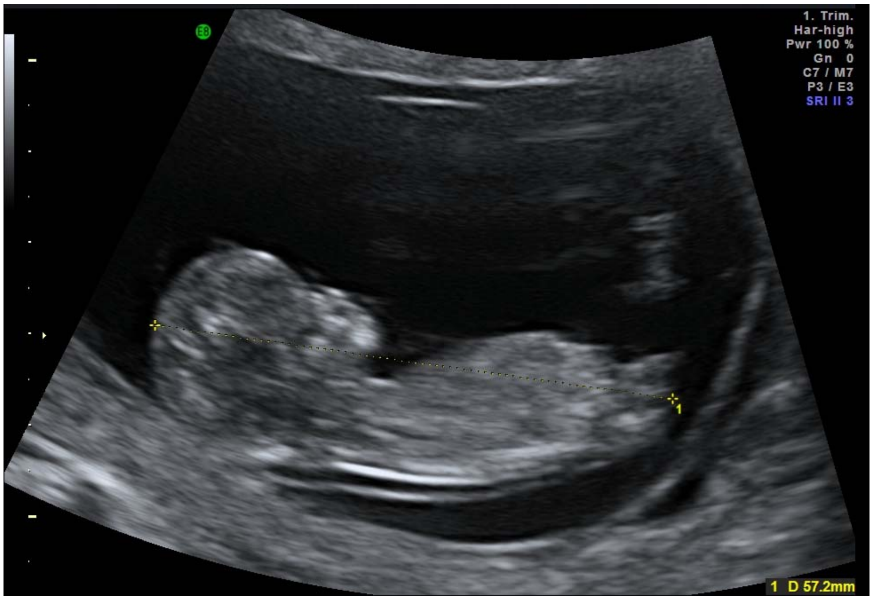

CORRECT:

- *Mid-sagittal section*
- *Horizontal position*
- *Neutral position*
- *Good magnification*
- *correct caliper position*

✓

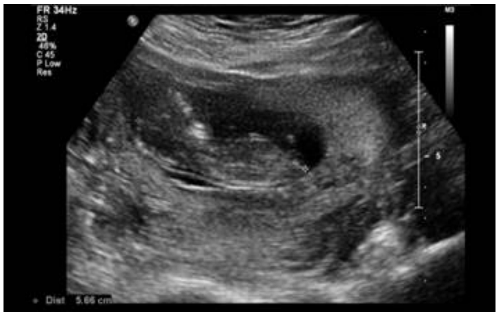

INCORRECT:

The section is not mid-sagittal

Magnification is poor

✗

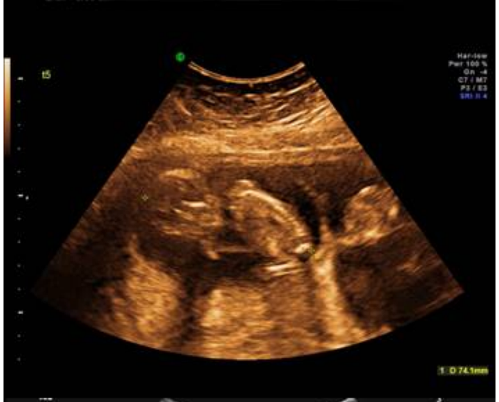

The section is not mid-sagittal

The fetus is flexed (not in a neutral position)

(It is also a twin pregnancy!)

✗

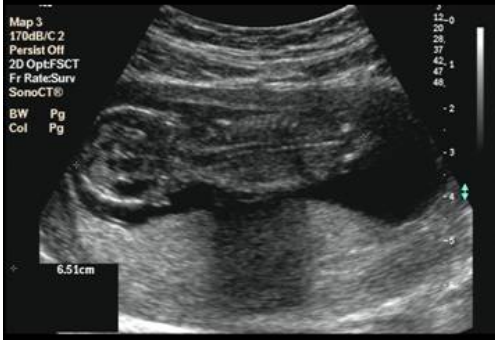

The section is not mid-sagittal:

You can see the spine in the middle - this is a coronal section

✗

Note: *Once you have saved an image the image memory icon in the lower right section of the screen changes to show the image was saved. To review individual images, press the patient key, select stored images from the on-screen menu and select review images.*

Head measurements

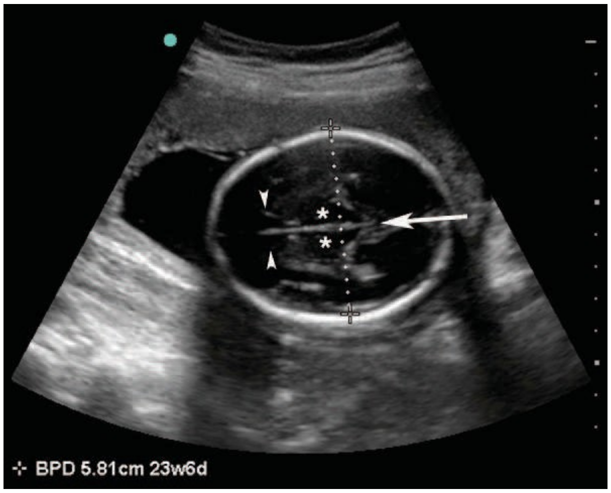

Note the cavum septi pellucidi (arrowheads), falx cerebri (arrow) and thalami (*)

Step 1: Obtaining the image

- Get a cross-sectional view of the fetal head at the level of the thalami and cavum septum pellicidum (CSP).
- As close as possible to the horizontal (angle of insonation as close as possible to 90°).
- Oval shape.
- Symmetrical.
- Centrally positioned, continuous midline echo (falx cerebri).
- The midline echo is broken anteriorly at one third of its length by the cavum septum pellucidum.
- The thalami should be located symmetrically on each side of the midline.
- The cross section of the fetal head should fill at least 30% of the monitor.

Step 2: Placing the calipers

- BPD: The intersection of the calipers should be placed on the outer border of the near skull border to inner edge of the far skull border (‘outer to inner’) at the widest part of the skull.

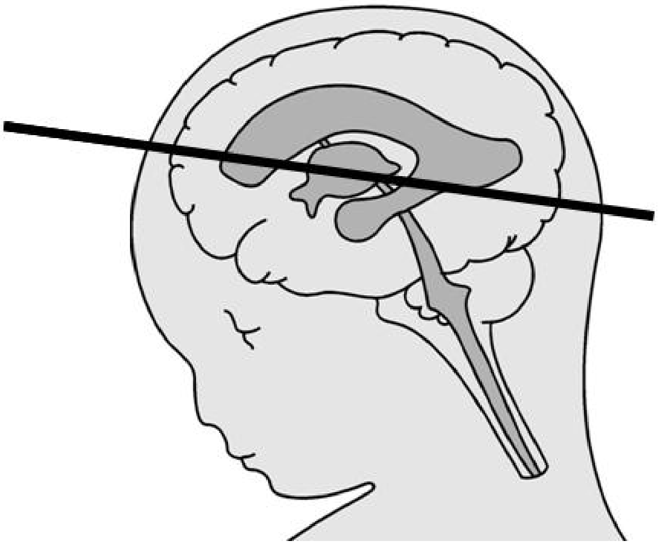

The level of the cross-section through the fetal head for correct measurement.

To measure the biparietal diameter (BPD)

1. Look for the fetal head and position the probe so that the head appears in the center of the screen with the transverse line dissecting the head uniformly. You should be able to see two bean shape structures on either side of the midline (thalami) and a small midline slit (cavum).
2. Once the image is frozen, select the M**easure** key.
3. Place the calipers **X** so that the line is perpendicular (90° angle) with the transverse line at the level of the thalamus.
4. Measure the distance between the **outer** wall to **inner** wall of skull bone.
5. Then press **Select** and **Enter** key.
6. Save the image using the **Save** key. 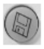

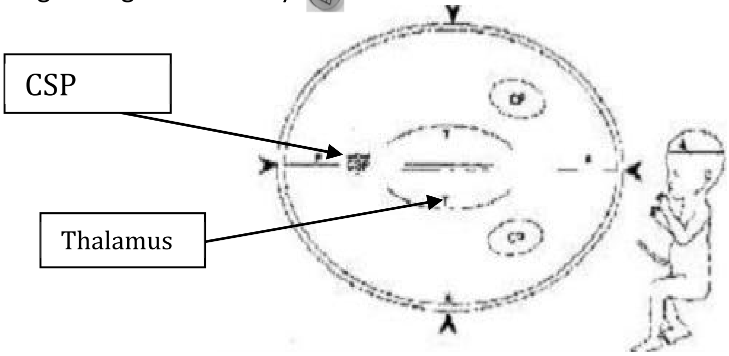

To measure the head circumference (HC)

1. Keep the same image and select HC using the **Arrow** keys at the bottom of the screen.
2. Adjust the circle so it covers the **outer** line of the skull and press the **Select** key followed by **Enter** key.
3. Save the image using the **Save** key. 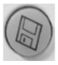

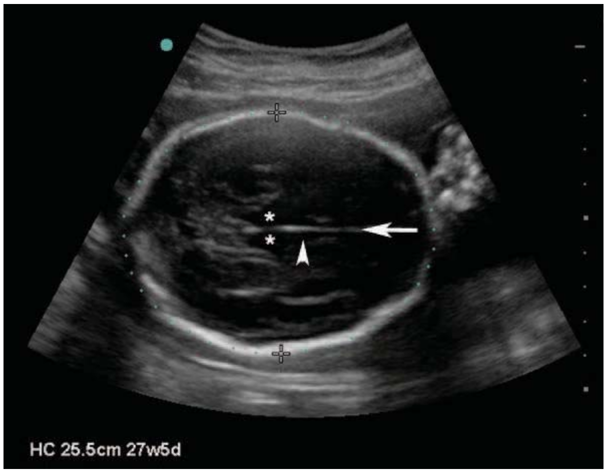

Head circumference measurement with thalamus (*), third ventricle (arrowhead), falx cerebri (arrow) and calipers measuring outer edge of the skull bone

When using the ellipse facility this should run along the outer border of the skull. Do not include the skin.

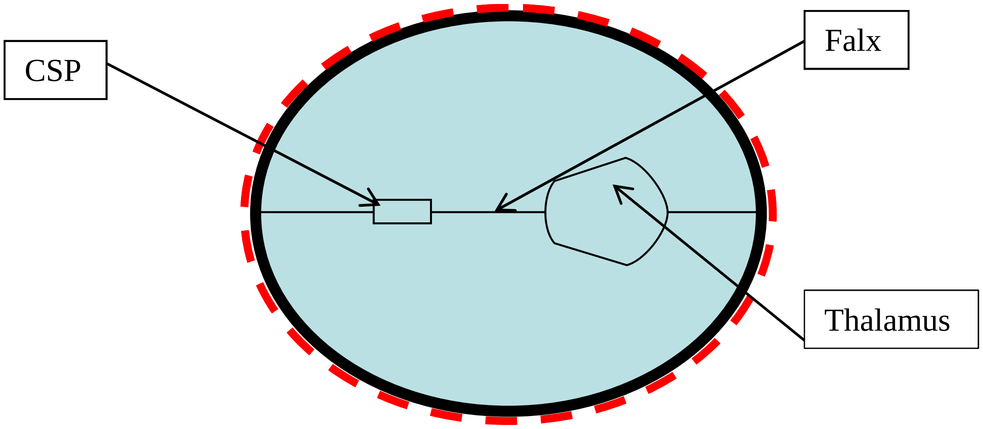

COMMON ERRORS:

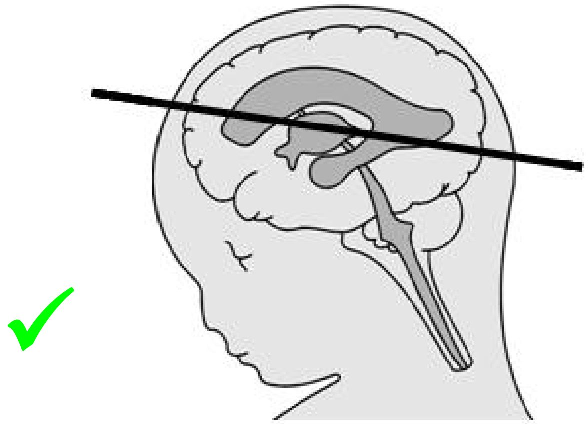

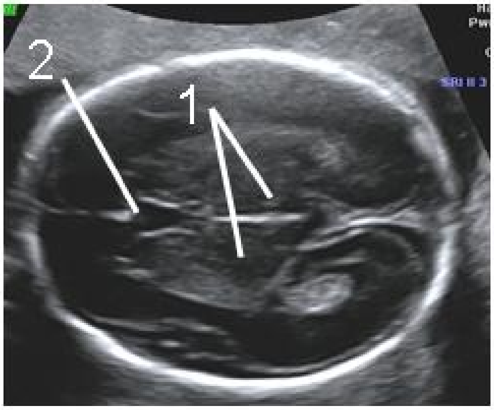

CORRECT: the main anatomic landmarks (1: thalami; 2: cavum septum pellucidum) are displayed.

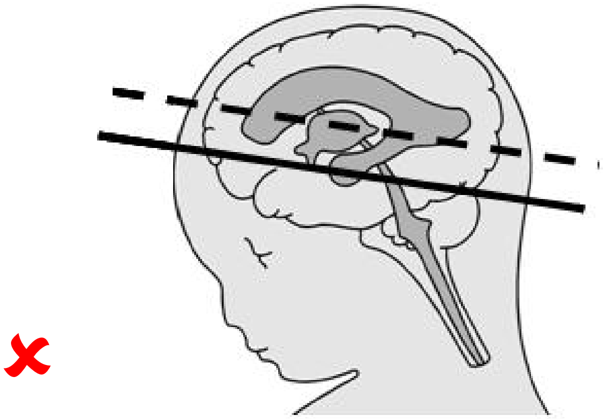

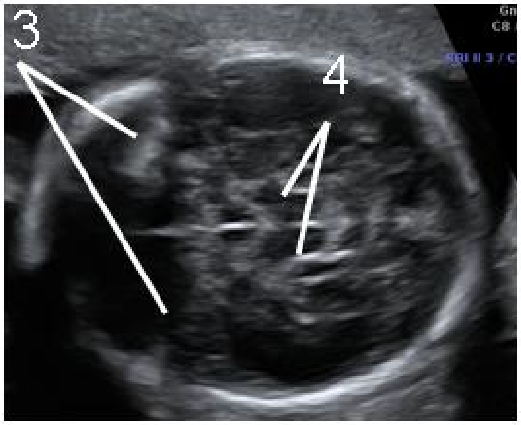

INCORRECT: the wings of the sphenoid bone (3) and cerebral peduncles (4) are demonstrated: this section is too low in the fetal head

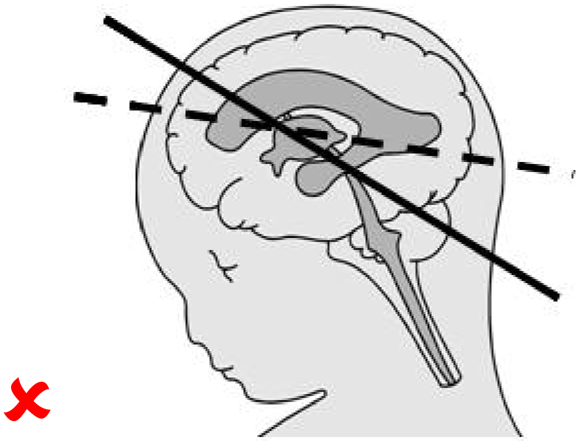

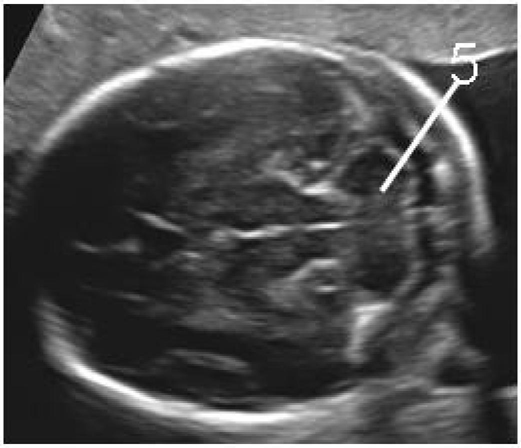

INCORRECT: the cerebellum (5) is demonstrated: this section is obtained along the suboccipitobregmatic plane and does not allow proper measurement of the occipitofrontal diameter. No: 1) Orbits, 2) Cerebellum, or 3) Cerebral pedundes.

SUMMARY:

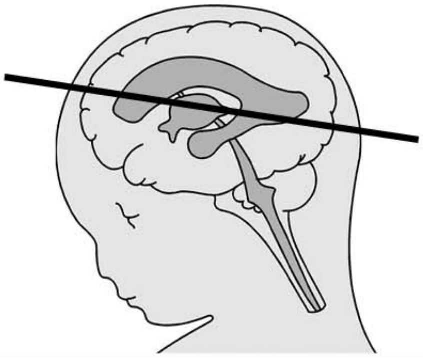

The main anatomic landmarks can be easily recognized between 14 weeks and term gestation.

Remember:

- **Level of the thalami**
- **Horizontal plane**
- **Oval shape**
- **Symmetrical**
- **Central position of falx cerebri**
- **Cavum septum pellucidum in anterior third**
- **Magnification**
- **Correct calliper placement**

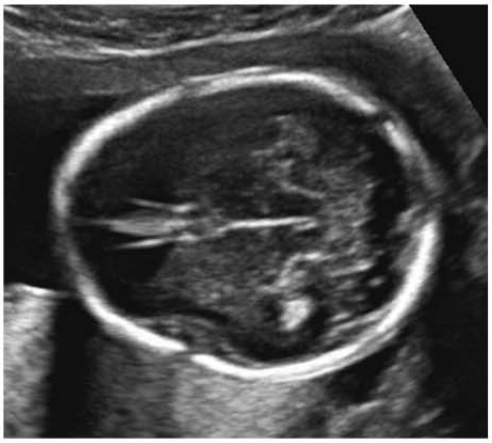

15 weeks

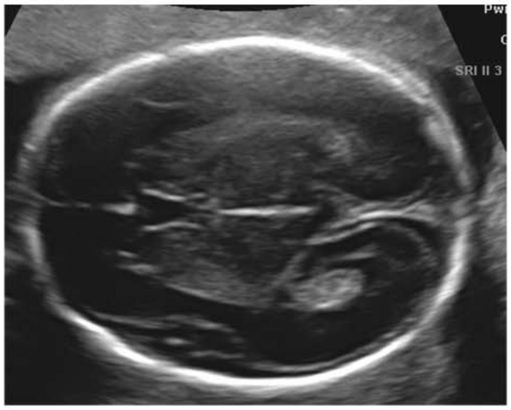

22 weeks

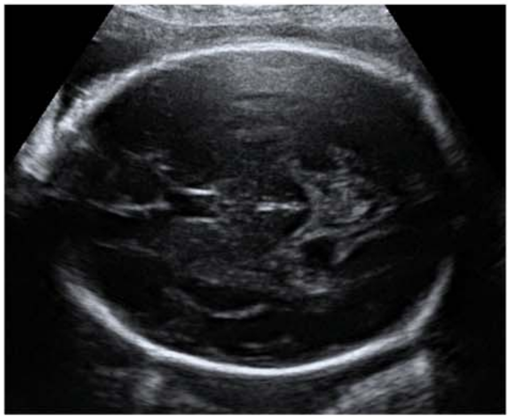

32 weeks

Abdominal measurements

To measure the abdominal circumference (AC) locate the abdomen and identify 3 abdominal structures: stomach, umbilical vein and spine. Focus the image so that it is in the center of the screen, the heart should not be visible for this measurement and the ribs should not appear (not be countable). When you have a satisfactory image freeze it and use the **Arrow** keys to select AC. Position the calipers at the opposite end of the abdomen and adjust the circle so it covers the whole circumference. Save the image using the **Save** key .

Abdominal Circumference (AC)

Step 1: Obtaining the image

- Transverse section of the fetal abdomen as close as possible to circular.
- Umbilical vein in its anterior third.
- Stomach bubble visible.
- Kidneys not visible.
- Bladder not visible.
- Magnify: The cross section of the fetal abdomen should fill at least 30% of the monitor screen.
- Ensure not to distort the circular shape of the fetal abdomen by applying too much pressure with the transducer.

Step 2: Placing the calipers

- The intersection of the calipers is placed on the outer borders of the body outline (skin covering).
- AP: from the posterior aspect (skin covering the spine) to the anterior abdominal wall.
- Transverse: at 90^0 to the AP, across the abdomen at the widest point.
- AC: Using the ellipse facility place the line of the ellipse on the outer border of the abdomen.

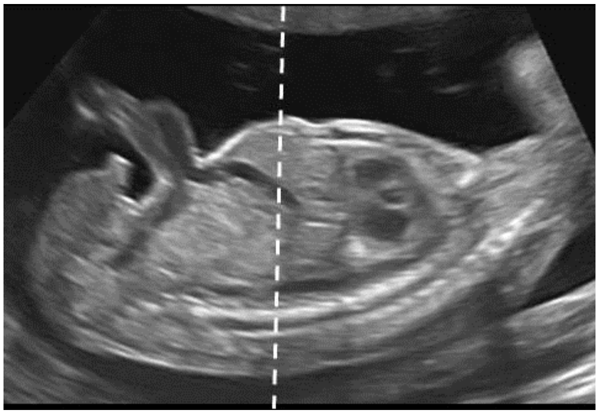

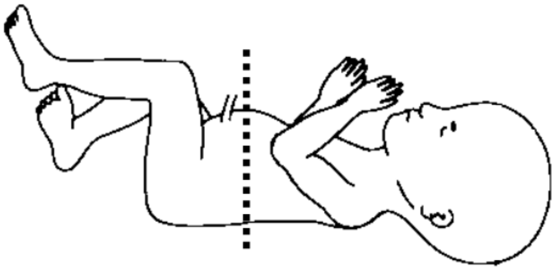

The level of cross-section through the abdomen for correct measurement

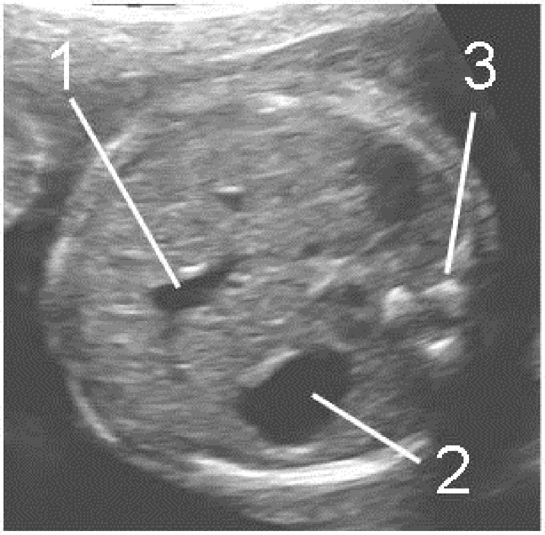

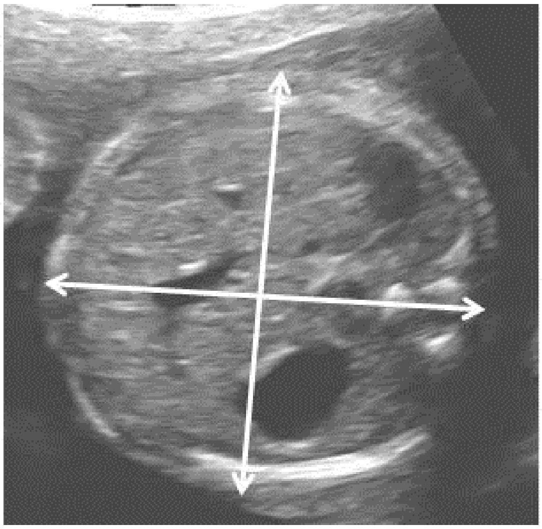

CORRECT:
-The image is well magnified
-The section is circular.
-The landmarks are seen:

1. Short segment of umbilical vein in the anterior third.
2. Stomach bubble visible.
3. Spine.

- The bladder and kidneys are not visible

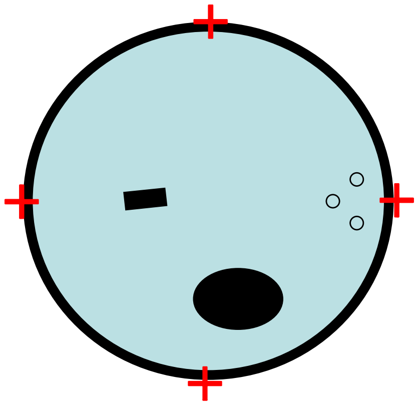

The calipers are positioned correctly, outer to outer.

When using the ellipse facility this should run along the outer border of the abdomen.

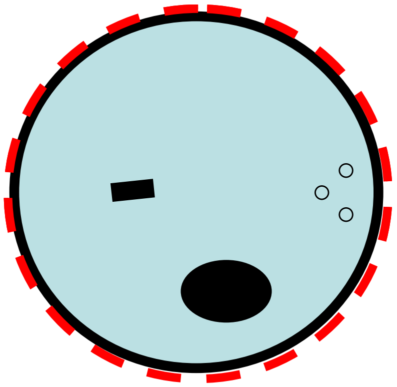

COMMON ERRORS:

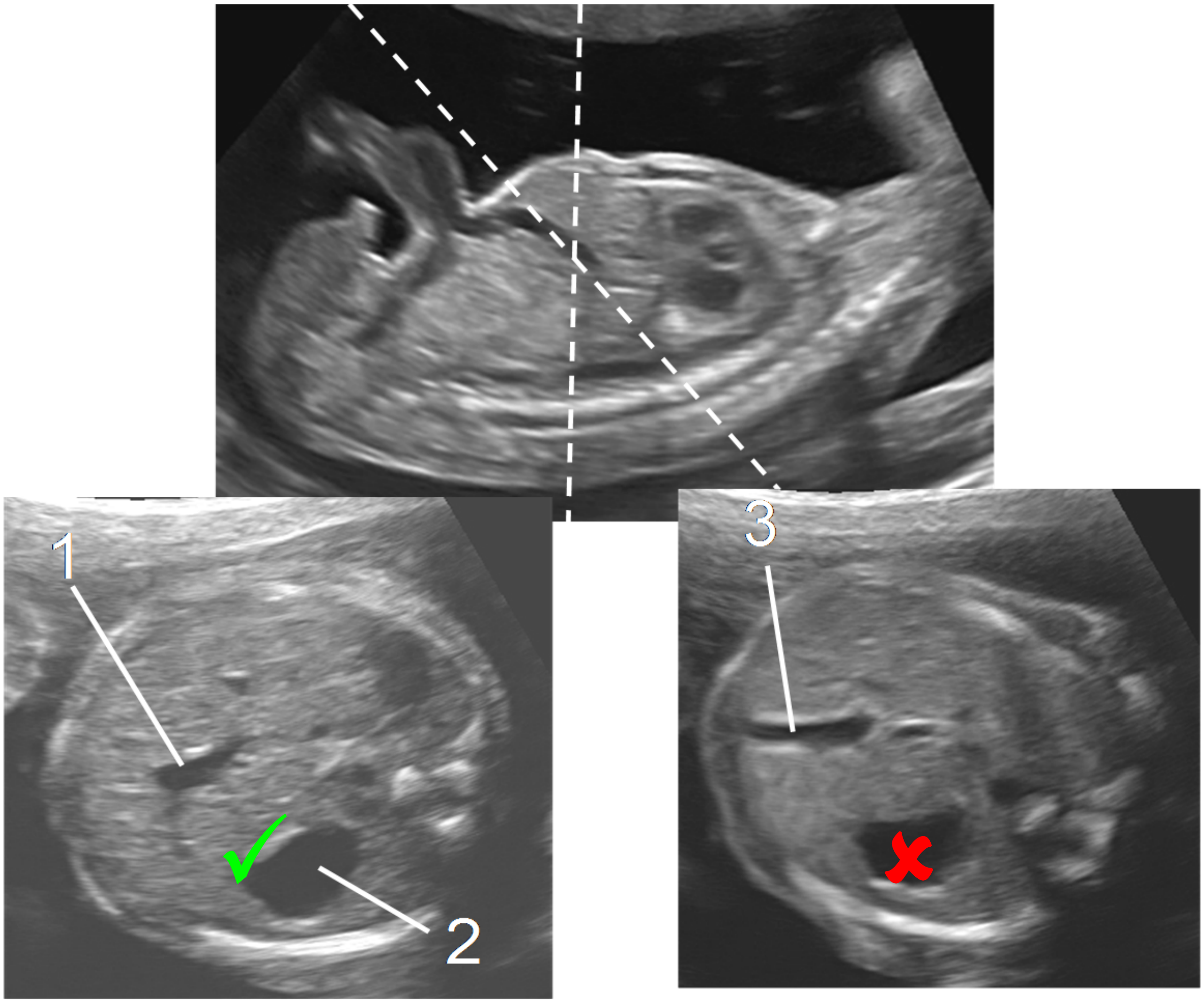

CORRECT:

-There is a short segment of umbilical vein in the anterior third (1), and the stomach is visible (2)

INCORRECT:

- The entire intra-abdominal umbilical vein (3) is demonstrated: this plane is too angled.

COMMON ERRORS:

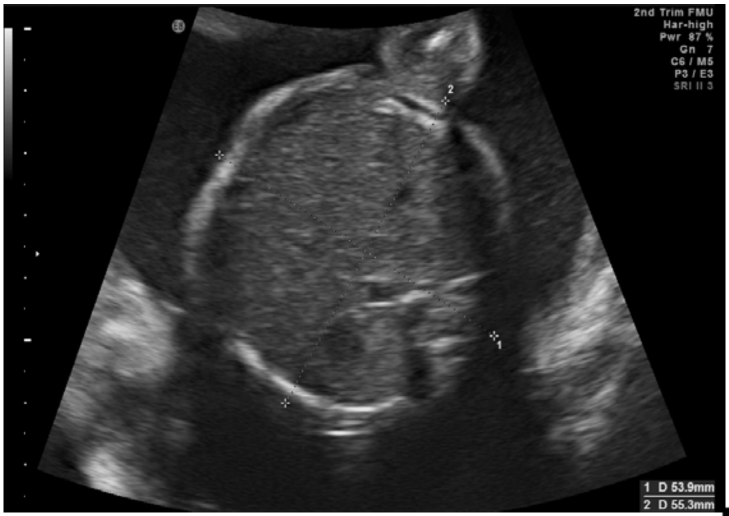

INCORRECT
- The stomach is NOT visible
- The umbilical vein is NOT visible.

INCORRECT
The umbilical vein is:
- Not in the anterior third
- Not a short segment

Like in the example above, the plane is too angled.
- Also, the stomach is not clearly visible

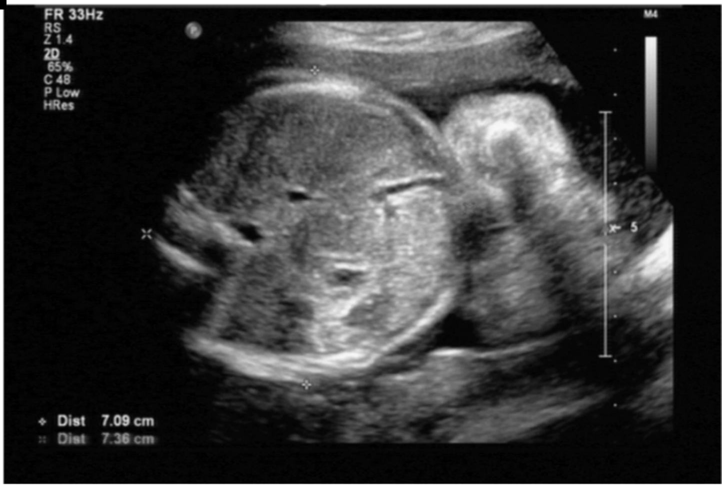

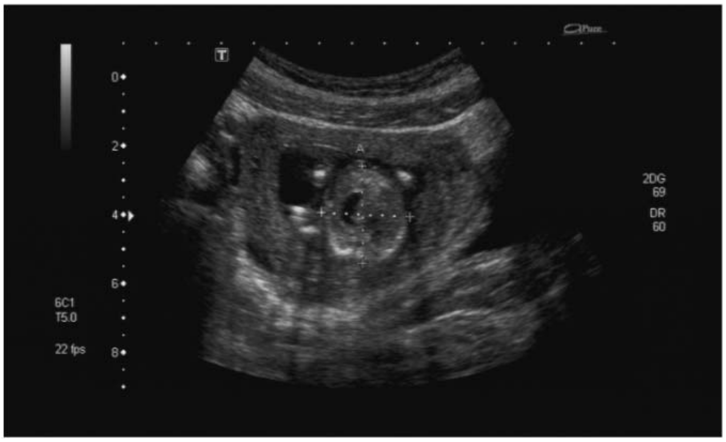

INCORRECT
- The magnification is very poor
- The umbilical vein may be correct but is not visible as the image is too small.

SUMMARY

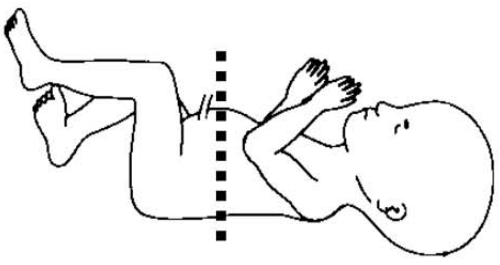

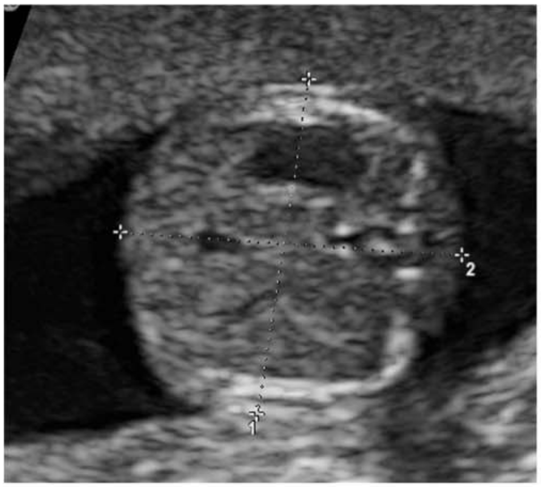

14 weeks

The main anatomic landmarks can be easily recognized between 14 weeks and term gestation.

Remember:

- As circular as possible
- Short umbilical vein in anterior third
- Stomach bubble visible
- Kidneys and bladder NOT visible
- Magnification
- Correct calliper placement

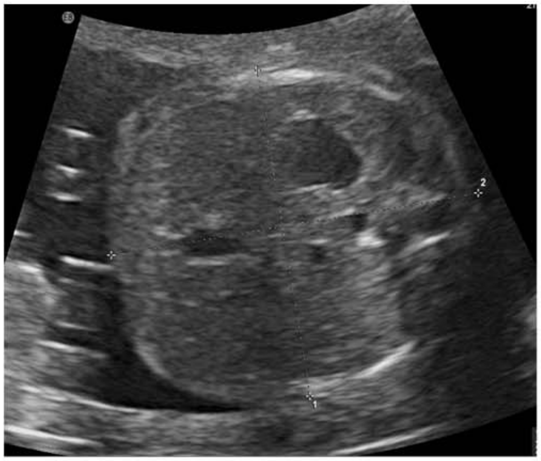

22 weeks

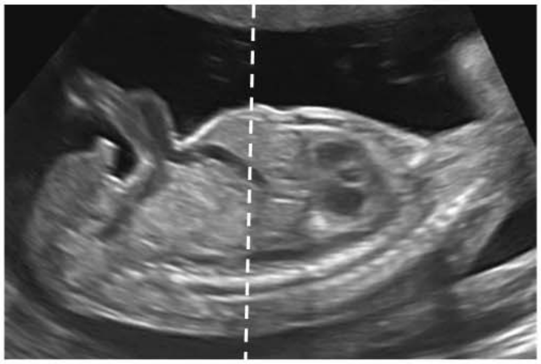

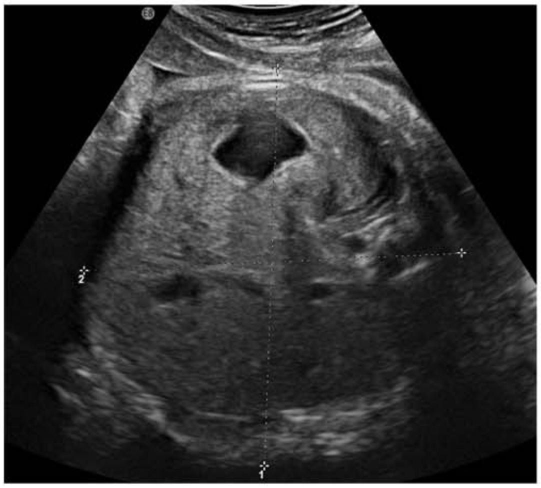

40 weeks

Femur length measurement

To measure the femur length (FL), locate the femur bone. If you can visualize 2, select the one closest to the probe (uppermost). When you have a satisfactory image freeze it and use the **Arrow** keys to select FL. Before positioning the calipers save the frozen image. Then on the same frozen image, position the calipers at the opposite end of the bone so it covers the entire length (without including the dark circle at the end of the bone). Press the **Select** key followed by **Enter** key. Save the image using the **Save** key.

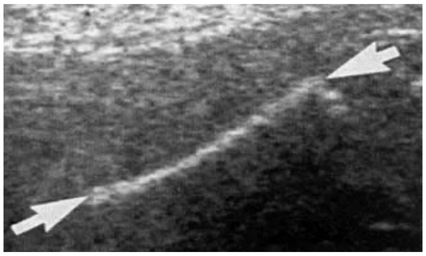

Femur Length

Step 1: Obtaining the image

- To be imaged as close as possible to the horizontal plane.
- Measure the bone closest to the probe.
- Angle of insonation of the ultrasound beam is 90°.
- The full length of the bone is visualized.
- The bone is not obscured by shadowing from adjacent bony parts.
- Magnify: The cross section of the femur should fill at least 30% of the monitor.

Step 2: Placing the calipers:

- The intersection of the calipers is placed on the outer borders of the edges of the femoral bone (outer to outer).
- The trochanter is not to be measured.

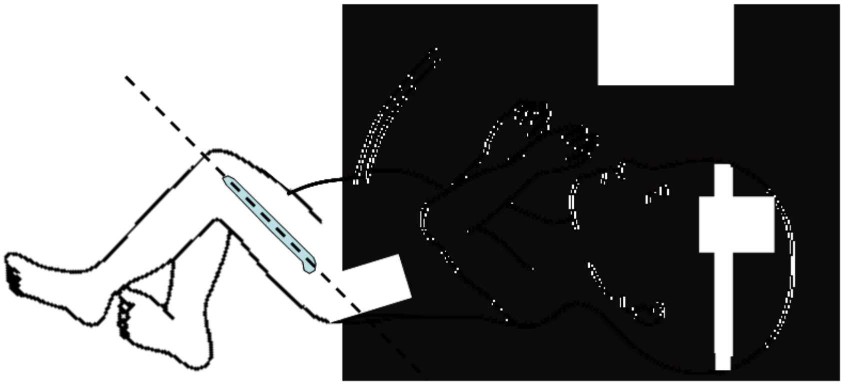

The level of the cross-section through the fetal femur for correct measurement.

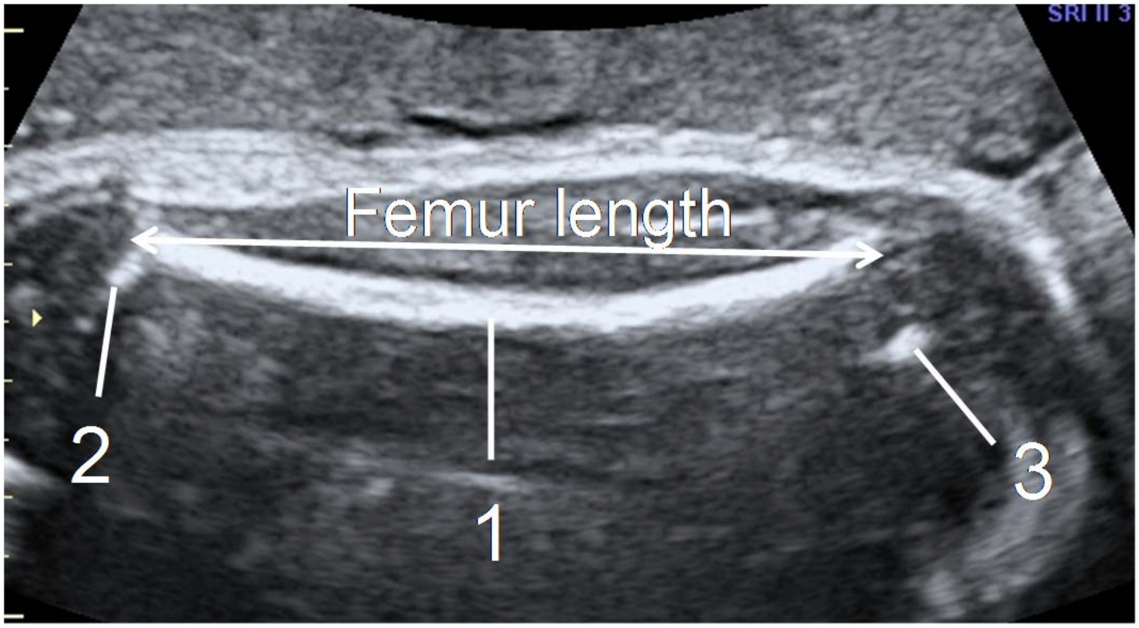

In the second trimester only the ossified diaphysis (1) of the femur can be demonstrated; in the third trimester the greater trochanter (2) and distal ossification center (3) can be seen and this allows one to orientate the section plane better.

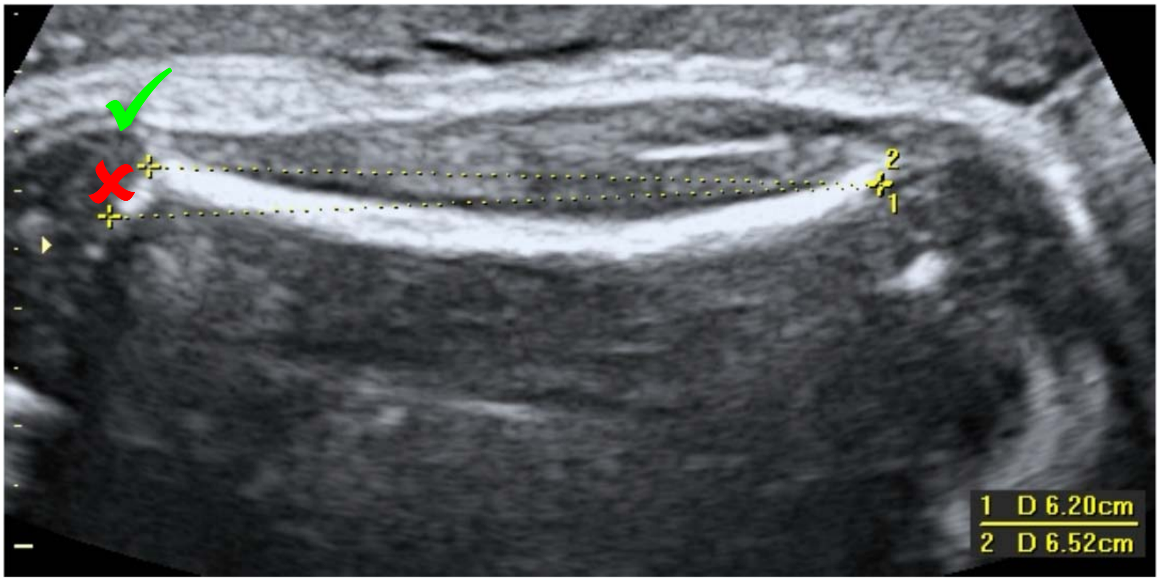

The greater trochanter should be avoided in measuring the femur length as this results in an excessive measurement (red X)

COMMON ERRORS:

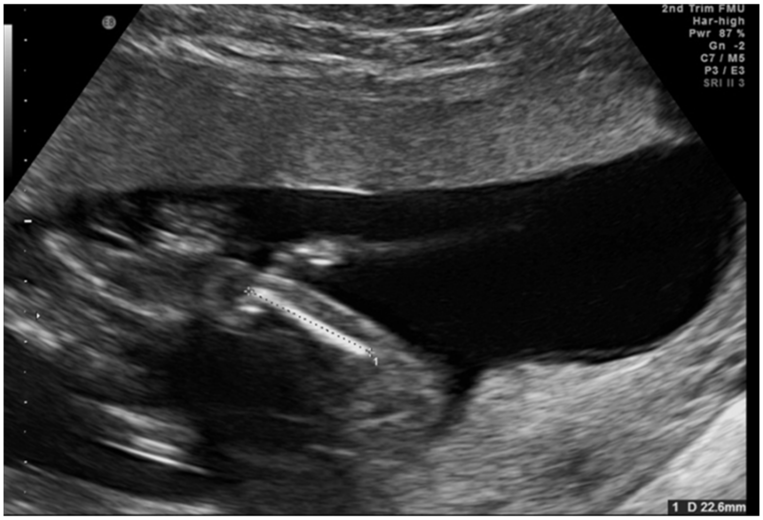

INCORRECT
The two edges of the femur are not clear. The main reason for this is poor magnification

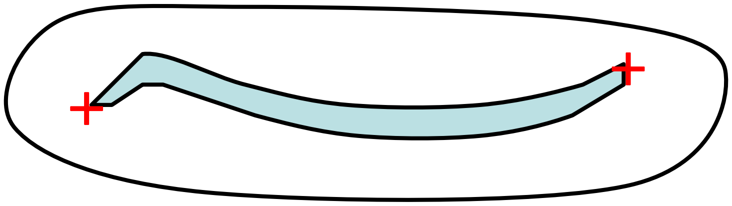

INCORRECT
The greater trochanter should not be included in the femur length

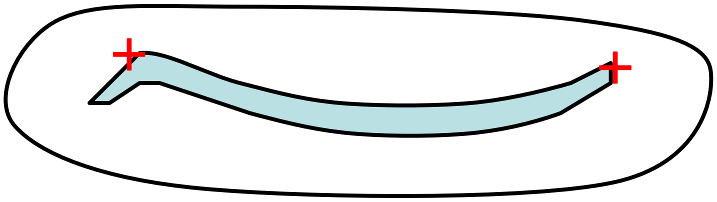

CORRECT

SUMMARY:

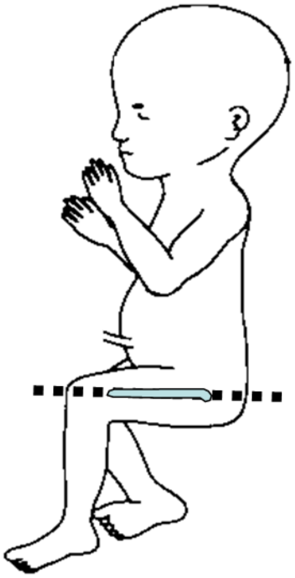

The femur can be easily recognized between 14 weeks and term gestation

Remember:

Horizontal as possible

Measure the bone closest to the probe.

See the full length of the bone

The bone is not obscured by shadows

Magnification

Correct calliper placement

Do not include greater trochanter

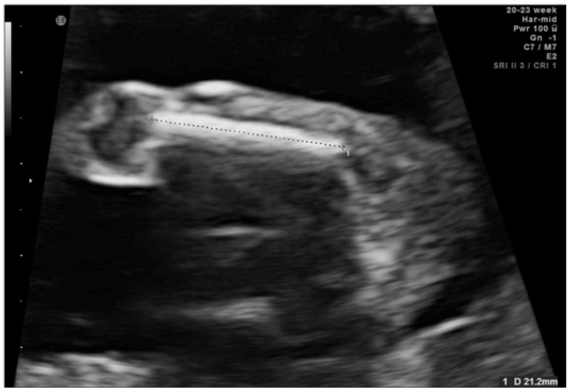

15 weeks

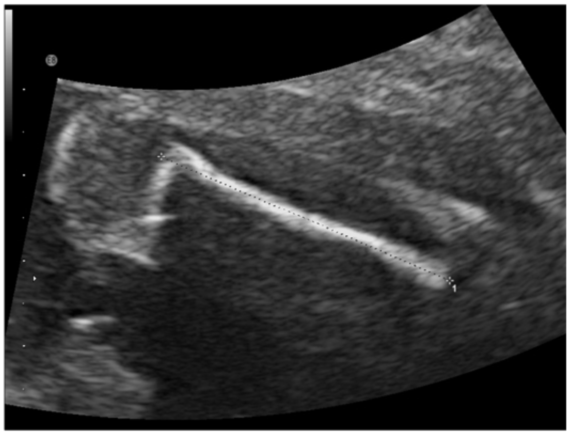

26 weeks

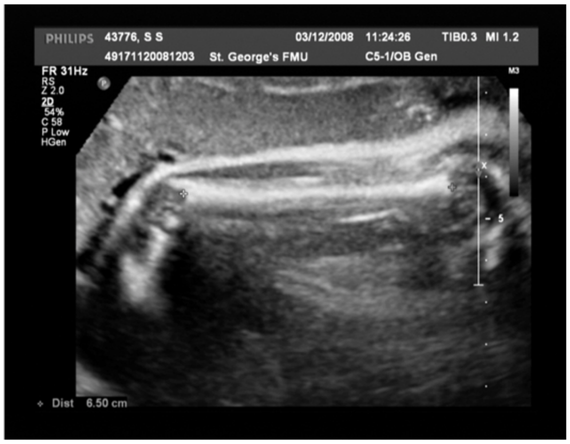

38 weeks

Interpretation

Keep in mind that the accuracy of gestational age assessment by ultrasound is the greater the earlier in pregnancy the examination takes place. A rough guide for the precision of gestational age estimation based on timing of the exam is listed below:

**Timing of examination**	**Precision**
6-12 weeks	+/- 3 days
13-20 weeks	+/- 1 week
21-24 weeks	+/- 10 days
25-28 weeks	+/- 2 weeks
> 28 weeks	+/- 3 weeks

Finishing the exam

1. Wipe the rest of the gel from the patient's abdomen and give her tissue to clean the rest herself. Wipe the transducer using clean paper and place it securely.
2. After finishing all measurements press the **patient** key and select patient report
3. Tell the patient what you have found (fetal heart, gestational age, multiple or single fetus, presentation of the placenta (particularly for placenta previa), position of the baby) and reassure her that the baby is doing well.
